# Leveraging Genomic Annotations and Pleiotropic Enrichment for Improved Replication Rates in Schizophrenia GWAS

**DOI:** 10.1371/journal.pgen.1005803

**Published:** 2016-01-25

**Authors:** Yunpeng Wang, Wesley K. Thompson, Andrew J. Schork, Dominic Holland, Chi-Hua Chen, Francesco Bettella, Rahul S. Desikan, Wen Li, Aree Witoelar, Verena Zuber, Anna Devor, Bipolar Disorder and Schizophrenia Working Group of the Psychiatric Genomics Consortium, Enhancing Neuro Imaging Genetics through Meta Analysis Consortium, Markus M. Nöthen, Marcella Rietschel, Qiang Chen, Thomas Werge, Sven Cichon, Daniel R. Weinberger, Srdjan Djurovic, Michael O’Donovan, Peter M. Visscher, Ole A. Andreassen, Anders M. Dale

**Affiliations:** 1 NORMENT, KG Jebsen Centre for Psychosis Research, Institute of Clinical Medicine, University of Oslo, Oslo, Norway; 2 Division of Mental Health and Addiction, Oslo University Hospital, Oslo, Norway; 3 Department of Neurosciences, University of California, San Diego, La Jolla, California, United States of America; 4 Multimodal Imaging Laboratory, University of California at San Diego, La Jolla, California, United States of America; 5 Department of Psychiatry, University of California, San Diego, La Jolla, California, United States of America; 6 Department of Cognitive Sciences, University of California at San Diego, La Jolla, California, United States of America; 7 Department of Radiology, University of California, San Diego, La Jolla, California, United States of America; 8 Institute of Human Genetics, University of Bonn, Bonn, Germany; 9 Department of Genetic Epidemiology in Psychiatry, Central Institute of Mental Health, Mannheim, Germany; 10 Lieber Institute for Brain Development, Baltimore, Maryland, United States of America; 11 Institute of Biological Psychiatry, MHC, Sct. Hans Hospital and University of Copenhagen, Copenhagen, Denmark; 12 Department of Biomedicine, University of Basel, Basel, Switzerland; 13 Department of Medical Genetics, Oslo University Hospital, Oslo, Norway; 14 MRC Centre for Neuropsychiatric Genetics and Genomics, School of Medicine, Cardiff University, Heath Park, Cardiff, United Kingdom; 15 The Queensland Brain Institute, The University of Queensland, Brisbane, Australia; 16 University of Queensland Diamantina Institute, University of Queensland, Translational Research Institute (TRI), Brisbane, Australia; Georgia Institute of Technology, UNITED STATES

## Abstract

Most of the genetic architecture of schizophrenia (SCZ) has not yet been identified. Here, we apply a novel statistical algorithm called Covariate-Modulated Mixture Modeling (CM3), which incorporates auxiliary information (heterozygosity, total linkage disequilibrium, genomic annotations, pleiotropy) for each single nucleotide polymorphism (SNP) to enable more accurate estimation of replication probabilities, conditional on the observed test statistic (“z-score”) of the SNP. We use a multiple logistic regression on z-scores to combine information from auxiliary information to derive a “relative enrichment score” for each SNP. For each stratum of these relative enrichment scores, we obtain nonparametric estimates of posterior expected test statistics and replication probabilities as a function of discovery z-scores, using a resampling-based approach that repeatedly and randomly partitions meta-analysis sub-studies into training and replication samples. We fit a scale mixture of two Gaussians model to each stratum, obtaining parameter estimates that minimize the sum of squared differences of the scale-mixture model with the stratified nonparametric estimates. We apply this approach to the recent genome-wide association study (GWAS) of SCZ (n = 82,315), obtaining a good fit between the model-based and observed effect sizes and replication probabilities. We observed that SNPs with low enrichment scores replicate with a lower probability than SNPs with high enrichment scores even when both they are genome-wide significant (p < 5x10^-8^). There were 693 and 219 independent loci with model-based replication rates ≥80% and ≥90%, respectively. Compared to analyses not incorporating relative enrichment scores, CM3 increased out-of-sample yield for SNPs that replicate at a given rate. This demonstrates that replication probabilities can be more accurately estimated using prior enrichment information with CM3.

## Author Summary

Genome-wide association studies (GWAS) have thus far identified only a small fraction of the heritability of common complex disorders, such as schizophrenia. Here, we demonstrate that by using auxiliary information we can improve estimates of replication probabilities from GWAS summary statistics. The proposed *Covariate-Modulated Mixture Model* (CM3) incorporates auxiliary information to construct an “enrichment score” for each single nucleotide polymorphism (SNP). We show that a scale mixture of two Gaussians provides a good fit to the observed effect size distribution stratified by the predicted enrichment score when applied the method to a recent genome-wide association study (GWAS) of SCZ (n = 82,315). Compared to estimates performed not using auxiliary information, the CM3 more accurately models the observed replication rates by stratifying on covariate-modulated enrichment scores. We observed that SNPs with low enrichment scores replicate with a lower probability compared to SNPs with high enrichment scores, even when both are genome-wide significant (p < 5x10^-8^). At model-based replication rates ≥80% and ≥90% there were 693 and 219 independent loci, respectively. Increased out-of-sample yield for SNPs ranked according to CM3 demonstrate the utility of incorporating auxiliary information via CM3.

## Introduction

Schizophrenia (SCZ) is one of the most heritable of human diseases, with estimates of the proportion of disease risk due to genetic factors ranging from 0.6 to 0.8[1]. However, until recently, GWAS have identified only a small number of associated genes or loci, accounting for a miniscule fraction of the heritability[2]. The turning point has been the establishment of the Psychiatric Genomic Consortium (PGC)[3], which has enabled the pooling of large numbers of independent studies, thus greatly increasing the power for identification of genes affecting disease risk, and confirming the polygenic nature of schizophrenia and other psychiatric disorders[2].

In most highly polygenic traits and diseases, individual genetic loci account for a very small portion of the phenotypic variance[4]. While increasing GWAS sample sizes is crucial, another key to improving estimates of which loci will replicate in independent studies is the application of statistical methods that incorporate auxiliary information. We have previously shown, using GWAS summary statistics from a smaller SCZ study (n = 21,856)[2], that genomic annotation categories[5, 6] and association with bipolar disorder (BIP)[7] significantly enriches test statistics for non-null associations. Pleiotropic enrichment was also observed between SCZ and other psychiatric and somatic phenotypes [8, 9]. Together with the ENCODE findings[10], these results provide a strong evidence against *a priori* equivalence, or statistical exchangeability, of all SNPs. These results instead suggest that the probability of association should be allowed to vary as a function of the relative enrichment of different SNP categories.

Here we present a novel algorithm, termed *Covariate Modulated Mixture Modeling* (CM3) that combines multiple sources of enrichment information to estimate SNP posterior effect sizes and to rank genetic loci based on covariate-modulated strength of association with a given trait or disease, i.e., loci that have the highest model-based estimates of probability of replication. The proposed method models thresholded z-scores as a function of enrichment categories, via logistic regression, to estimate a relative enrichment score for each SNP. Enrichment scores are then stratified into K bins: for a given enrichment stratum, we fit a scale-mixture of two Gaussians model to summary statistics within the stratum. These stratified mixture models allow for estimation of the expected z-scores and replication rates, given the observed z-scores and the effective sample size of the discovery and replication datasets. We hypothesized that sorting SNPs by the predicted replication probability from CM3 would improve out-of-sample yield, for a given replication rate, relative to the standard approach of sorting SNPs by nominal p-values alone. Here we compute the empirical replication rate as the proportion of SNPs having p values≤ 0.05 in an independent sample within a set of SNPs.

We applied the CM3 method to the latest PGC SCZ sample, including n = 35,476 patients with SCZ and n = 46,839 controls, across 52 separate sub-studies[11]. We incorporated the following four auxiliary information categories: 1) linkage disequilibrium (LD)-weighted genomic annotations; 2) total LD (TLD); 3) heterozygosity (H); and 4) pleiotropy with bipolar disorder (BIP) (see Materials and Methods). Our results show that a stratified scale-mixture of two Gaussians model appears to fit the SCZ data well across different enrichment strata. After incorporating auxiliary information via CM3, more SNPs replicate at a given rate (e.g., 90%) compared to sorting SNPs by nominal p-values alone. Thus, enrichment methods such as CM3 may provide effective criteria for ranking SNPs in GWAS for further investigation, with potential implications for improved gene discovery and polygenic risk prediction for personalized medicine.

## Results

### Sources of Differential Enrichment

**Fig 1:**
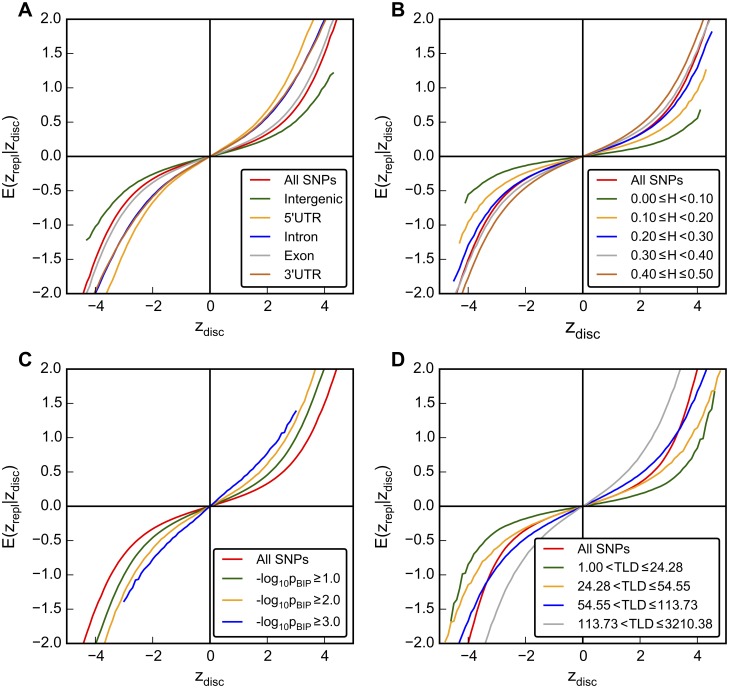
Mean replication z-scores stratified by genomic annotation, pleiotropy and heterozygosity. The conditional mean z-scores in replication sample (y axis) were plotted against the z-scores in the discovery sample (x axis). The shrinkage of replication z-score is differentiated by A.) genomic annotation categories (All SNPs; Intergenic; 5’ untranslated region,5’ UTR; Intron; Exon; and 3’ untranslated region, 3’ UTR), B.) by heterozygosity (H) intervals, C.) by associations with bipolar disorder (BIP; All SNPs; -log_10_ p ≥ 1.0; -log_10_ p ≥ 2.0; and log_10_ p ≥ 3.0) and D.) by total LD (TLD) intervals. All plots were generated by randomly assigning 26 of the PGC Schizophrenia sub-studies as discovery sample and 26 as replication sample (split half). The average value over 500 iterations is shown.

We show the results from 500 iterations of the resampling algorithm using split-halves (50% of PGC SCZ sub-studies as discovery and the other 50% as replication samples), with inverse-variance weighted meta-analysis z-scores computed for both “discovery” and “replication” samples[12]. Fig 1A shows the mean z-score across replications as a function of z-scores in the discovery samples, for different LD-weighted genomic annotation categories. For a given z-score in the discovery sample, tag SNPs in LD with enriched categories such as 5’UTR, exon, and 3’UTR variants have a higher mean z-scores in the replication sample compared to less enriched categories (e.g., intergenic; see S2 Fig for all categories studied). We also investigated the relative ‘‘enrichment” due to heterozygosity (H). Fig 1B shows mean replication z-scores as a function of z-scores in the discovery sample, for different ranges of H. For a given z-score in the discovery sample, tag SNPs with higher H have a higher mean z-score in the replication sample compared to SNPs with lower H. In addition, we calculated the mean replication sample z-scores as a function of z-scores in the discovery sample for different levels of association with BIP, after removing overlapping samples from the PGC BIP data[7]. For a given z-score in the discovery sample, SNPs with more significant association with BIP have a higher mean z-score in the replication sample compared to SNPs with less significant association with BIP (less enriched, Fig 1C). We also observed that the mean replication sample z-scores increases for a given discovery sample z-score as the total LD increases (Fig 1D). Taken together, this shows that, after conditioning on auxiliary information, SNP z-scores are not exchangeable in terms of association with SCZ. The properties of each SNP—LD with annotation categories (Fig 1A and S2 Fig), heterozygosity (H, Fig 1B and S7 Fig), association level with other traits (Fig 1C) and total LD (TLD, Fig 1D)—have implications regarding replicable associations with SCZ.

### Combined Differential Enrichment Score

**Fig 2:**
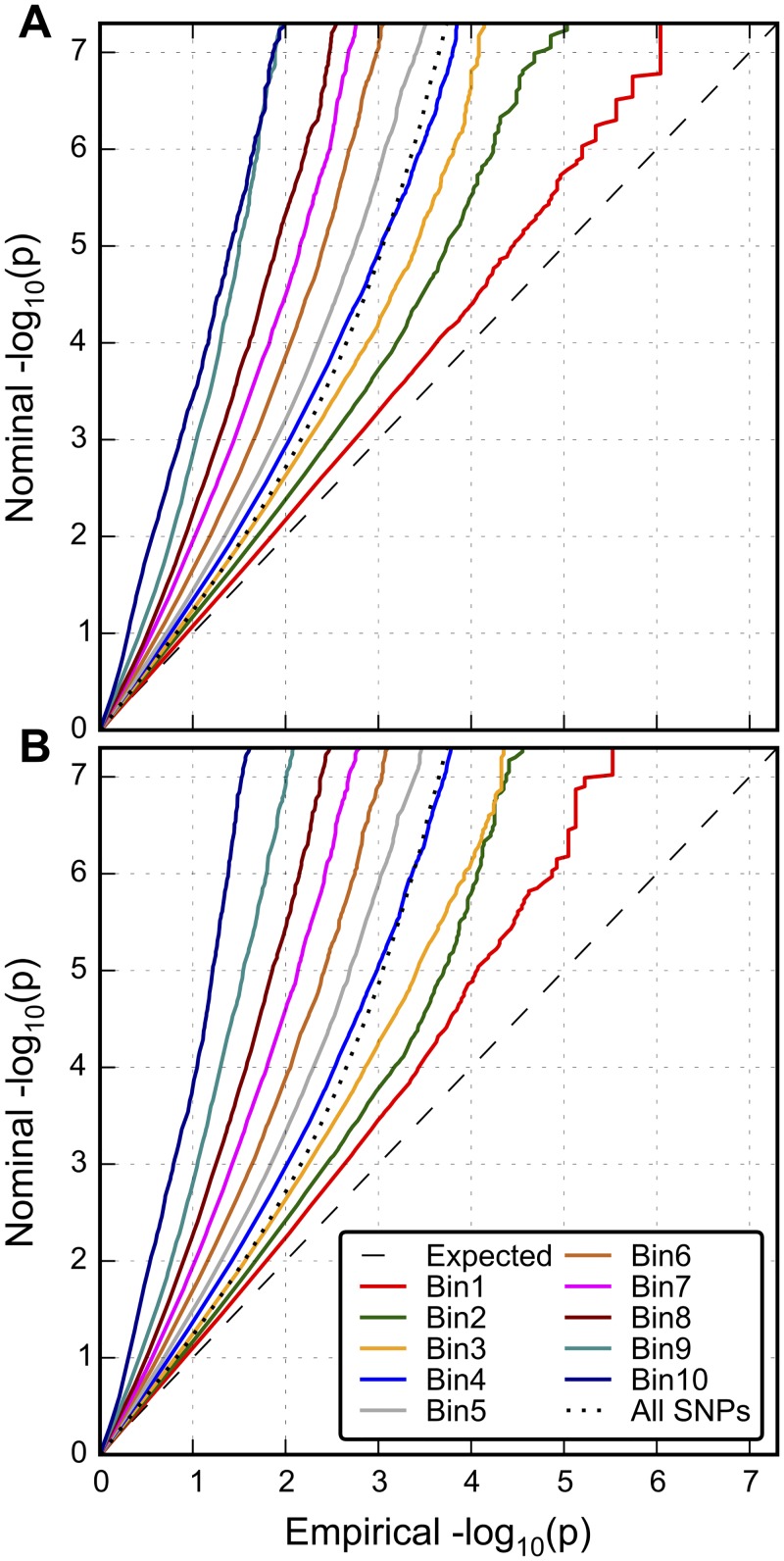
Enrichment of SNP associations with schizophrenia conditioned on predicted enrichment scores. The conditional Q-Q plot shows the enrichment of SNP association with schizophrenia stratified by predicted relative enrichment scores A.) based on LD-weighted Annotation categories, heterozygosity and total LD score and B.) based on LD-weight Annotation categories, heterozygosity, total LD score and SNP association with bipolar disorder. The predicted enrichment scores are equally divided into 10 disjoint intervals or bins (from the least enriched stratum, Bin1, to the most enriched stratum, Bin10). The dashed line indicates the null distribution and dotted line indicates all SNPs, i.e., not stratified. Different colors indicate different intervals of predicted enrichment scores. The leftward shift of the each curve compared to the null line indicates the relative enrichment. SNPs in the MHC region were excluded and then pruned based on the LD structure from the 1000 Genomes European subpopulation at r^2^ < 0.8.

**Fig 3:**
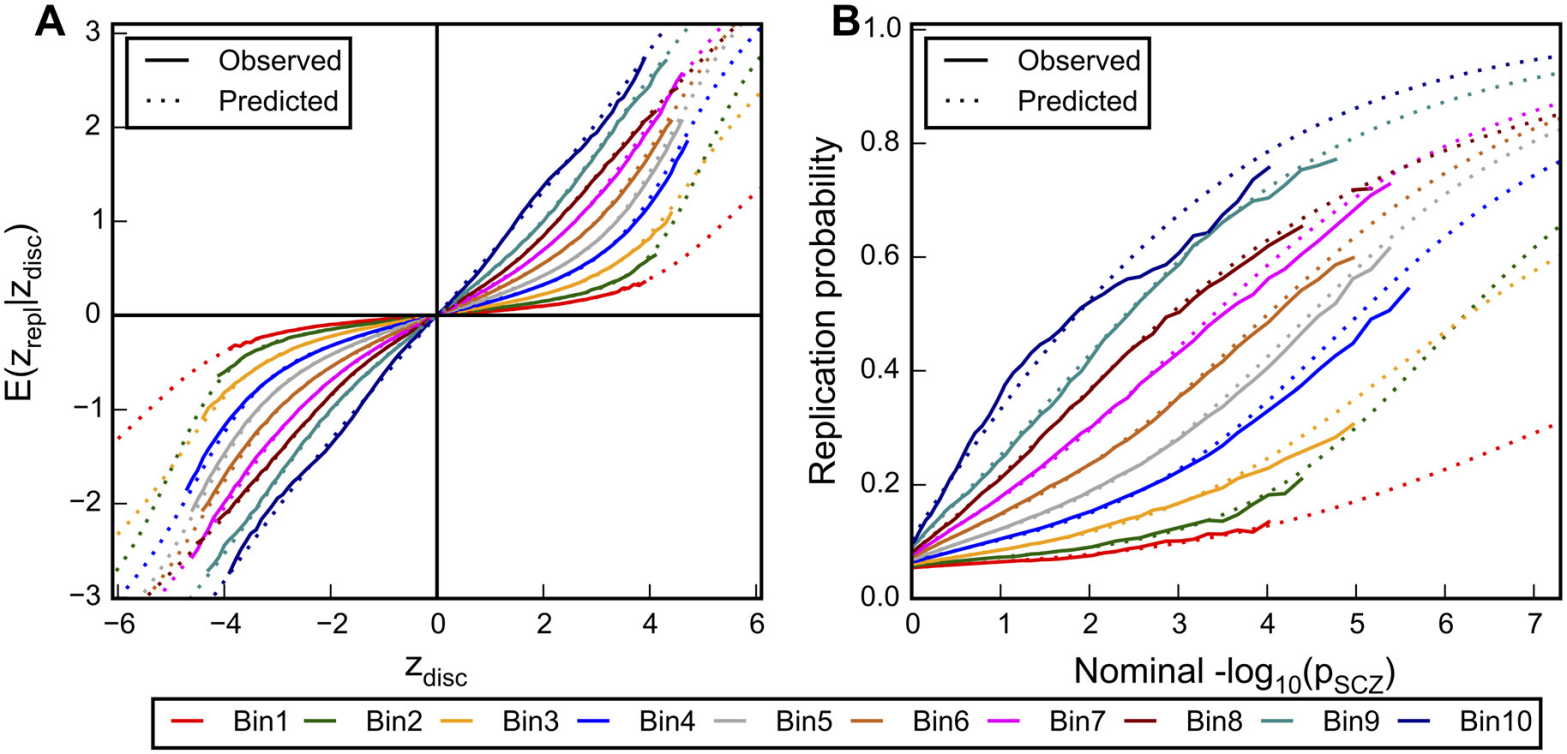
Mean replication z-score and replication rate stratified by enrichment scores. A.) The observed (solid lines) and predicted (dotted lines) mean z-scores in the replication sample (y axis) were plotted against the z-scores in the discovery sample (x axis). The shrinkage of replication z-scores is differentiated by disjoint intervals of relative enrichment scores. B.) The observed (solid lines) and predicted (dotted lines) replication probabilities were plotted against the negative common logarithm of nominal p values of schizophrenia SNPs in discovery sample (x axis). Colors indicate the 10 disjoint intervals of relative enrichment scores, ranging from the least enriched (Bin1) to the most enriched (Bin10). All data were generated by randomly assigning 26 of the PGC schizophrenia sub-studies as discovery sample and 26 as replication sample (split half). The averaged value over 500 iterations was shown.

We combined information from the different sources of auxiliary information (LD-weighted annotation categories, TLD, BIP, and H) using a multiple logistic regression model, to compute the predicted relative enrichment score for each SNP (see Materials and Methods). The relative enrichment scores of all SNPs were stratified into ten equally spaced disjoint intervals. Fig 2 shows the conditional Q-Q plots displaying the distribution of summary statistics for the PGC SCZ conditional on different levels of enrichment, from the least enriched stratum (Bin 1) to the most enriched stratum (Bin 10). Q-Q curves are thresholded at–log_10_ p ≤ 7.3 to focus on SNPs below genome-wide significance. Comparing Fig 2A with Fig 2B shows that by including pleiotropy with Bipolar Disorder (BIP) as extra source of auxiliary information the level of enrichment increases. Fig 3A shows the stratified discovery and replication observed mean z-scores of these strata (solid lines) with split half samples, using the resampling-based strategy (see Materials and Methods). (The results for other re-sampling proportions are shown in S3 Fig). The shape of the posterior z-score functions (Fig 3A), monotonically increasing but with a relatively flat region in the middle, is characteristic of mixture distributions, with non-linear “shrinkage” towards zero (see Efron [13]). For a given z-score in the “discovery” sample, the z-scores in the “replication” sample increases with increasing degree of predicted enrichment. For example, in the case of a SNP with a z-score in the discovery sample of 2, the expected z-score in the replication sample is approximately 0.10 for the least enriched category, and approximately 1.30 for SNPs in the most enriched strata.

The solid lines in Fig 3B show the mean observed replication probabilities across random split-half partitions as a function of nominal p-values in the discovery samples, for different enrichment strata (see Materials and Methods). Results for other training/replication partition proportions are shown in S4 Fig. As expected, Fig 3B shows an increase in observed replication probability with increased relative enrichment factor levels for a given p-value. For examples, for SNPs with a p-value of 0.001 (-log_10_(p) = 3.0) in the discovery sample, the observed replication rate is close to 0.09 in the least enriched stratum, and increased to an observed replication rate of 0.68 for the most enriched stratum. Related to this, for a given observed replication rate, the p-value varies dramatically across enrichment strata. The corresponding Figs illustrating the observed relationships of z^2^ between discovery and replication samples are shown in S5 Fig.

### Modeling Test Statistics and Replication Rate

To investigate if we can model the nonparametric estimates of replication test statistic means and variances (solid lines in Fig 3A and 3B), we fit a scale-mixture of two Gaussians model to each enrichment stratum (see Materials and Methods for details). The dotted lines in Fig 3A indicate the posterior mean z-scores in replication sample as function of z-scores in discovery sample for the different enrichment strata. The corresponding observed and predicted replication probability plots are shown in Fig 3B. Note that for SNPs satisfying the standard GWAS significance threshold (p < 5 x 10^−8^), the predicted replication rate ranges from close to 0.28 for the least enriched stratum, to 0.94 in the most enriched stratum. In other words, SNPs obtaining the commonly used p-value threshold in GWAS (p = 5x10^-8^) replicate more frequently if associated with a high relative enrichment score compared to SNPs with the same p-values but having a low enrichment score. The proposed mixture model appears to provide a good fit to the observed data across different enrichment levels and discovery and replication sample sizes. S3–S5 Figs show the model performance of other discovery/replication partition proportions.

**Fig 4:**
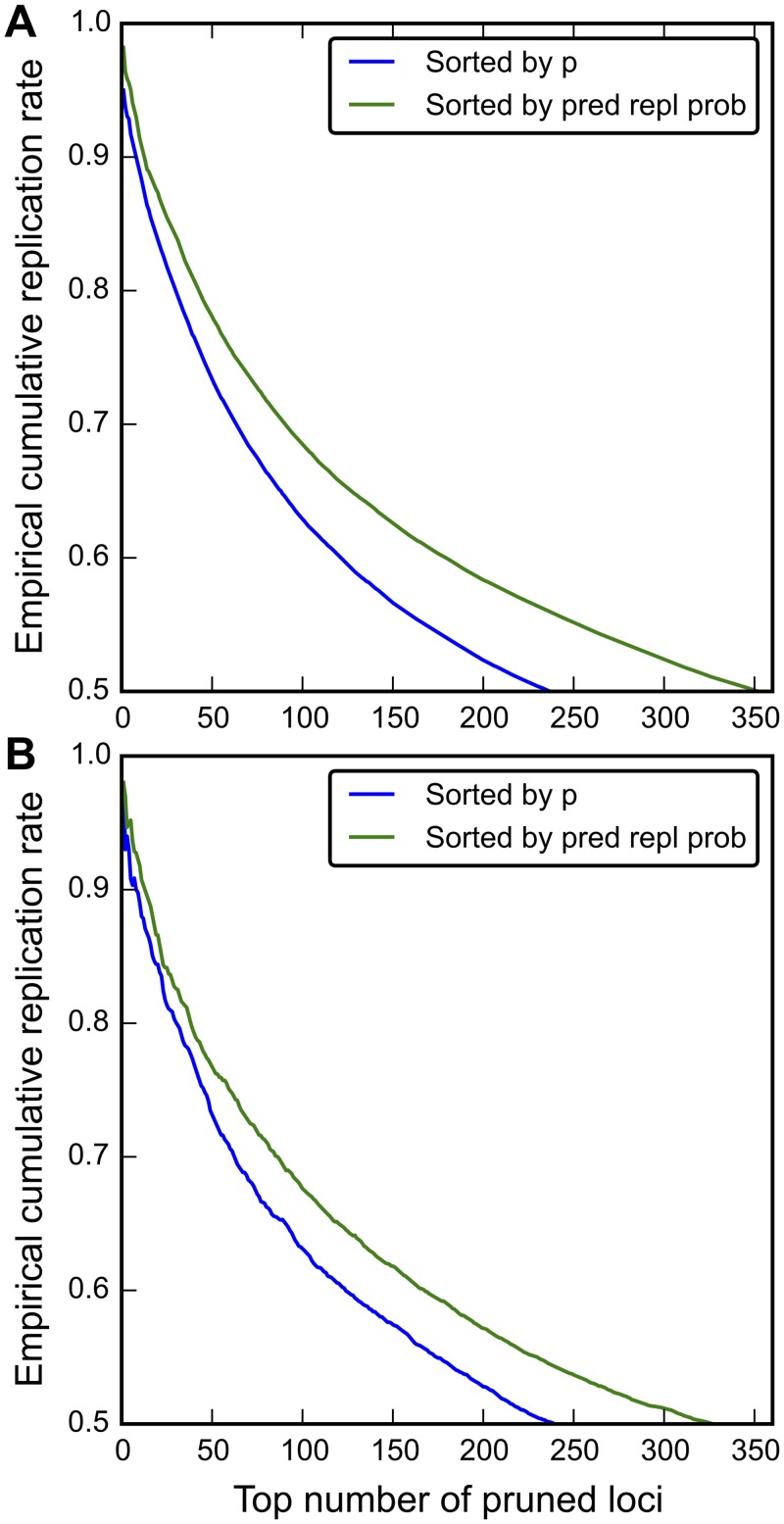
CM3 improves power of identifying gene loci. The average empirical cumulative replication rates (y axis) are plotted against the number of SNPs replicating at that rate > 0.5 (x axis), after removing MHC region SNPs and pruning at LD r^2^ < 0.1. A.) The full PGC sample was used to estimate predicted replication probability (pred repl prob). For each iteration, 26 PGC schizophrenia sub-studies were randomly assigned to the discovery sample, and the rest to the replication sample (split half). The average values over 500 iterations are shown, and B.) Half of the PGC sample (26 sub-studies) was used to estimate the predicted replication probability. For each iteration, 26 PGC schizophrenia sub-studies were randomly assigned to the discovery sample, and the rest to the replication sample (split half). Then, the predicted replication probability was estimated by applying the CM3 method on the discovery sample with 50 iterations. The p values (computed by meta-analysis) of the discovery sample and the predicted replication probabilities (computed by CM3) were used to sort SNPs in replication sample, consist of rest of the sub-studies. The average replication rates across 50 iterations were shown. Colors indicate different sorting criteria (green: sorted by prediction replication probability and blue sorted by nominal p values).

To investigate the effect of sorting SNPs based on predicted replication probability instead of by nominal p-value, we computed the cumulative empirical replication rate using the random partition approach (split-half). Fig 4 shows the comparison of the observed cumulative replication rate with SNPs sorted by the predicted replication probability from CM3 (from high to low) and nominal p-values (from low to high). Using the CM3 method, a larger number of loci are selected for a given cumulative observed replication rate than when ranking SNPs by nominal p-values. For instance, for the CM3 method an average of 353 loci replicate at a replication rate of 0.5, whereas an average of 238 replicate at the same rate when using nominal p-values without auxiliary information (Fig 4A). Further, when sorted by p-values, no SNPs replicate at a rate higher than 0.95, whereas when sorted by predicted replication probability, the highest-ranked SNPs replicate at a rate of 0.982 (Fig 4A). Fig 4B shows out-of sample performance of CM3, i.e., only the split half discovery sample was used to fit model parameters, compared to the nominal p-values based method. At a replication rate 0.5, 328 loci replicated sorted by predicted replication probability from the CM3 methods. Taken together, this shows that incorporating auxiliary information via CM3 can provide a larger yield of SNPs for a given observed replication rate.

To assign posterior effect size estimates and predicted replication probability to each SNP for the whole PGC SCZ sample, we computed a fine grid “lookup table” as a function of the observed z-scores in the discovery sample and the enrichment score (see Materials and Methods). S8 Fig compares sorting of SNPs based on the predicted replication probability vs. on nominal p-value. The change due to sorting by CM3 is most pronounced for SNPs having smaller effect sizes, i.e., larger p-values (upper right corner of S8 Fig) and less so for SNPs having smaller p-values (lower left corner of S8 Fig). We found 693 independent non-MHC loci (LD r^2^ < 0.1, clumped by distance 250kbp) having predicted replication probability ≥ 0.8, and 219 having predicted replication rate ≥ 0.9 (S1 Table). The predicted replication rate corresponding to the GWAS p-value threshold of 5x10^-8^ was 0.8571 in the split-half discovery/replication analysis, without stratification by relative enrichment scores (S9 Fig). At this estimated replication threshold, CM3 identified 9 more regions than p-value based method (Fig 4). CM3 performs better when the size of discovery sample is smaller than replication sample, given that overall sample size is fixed (S6 Fig). We also repeated the analysis without including BIP as enrichment sources. The number of clumped independent non-MHC loci with predicted replication probability > 0.8 (0.9) becomes 428 and 201. We display the predicted replication probability in the sixth column of S1 Table.

### Relative Importance of Auxiliary Information Categories Using Logistic Regression

**Fig 5:**
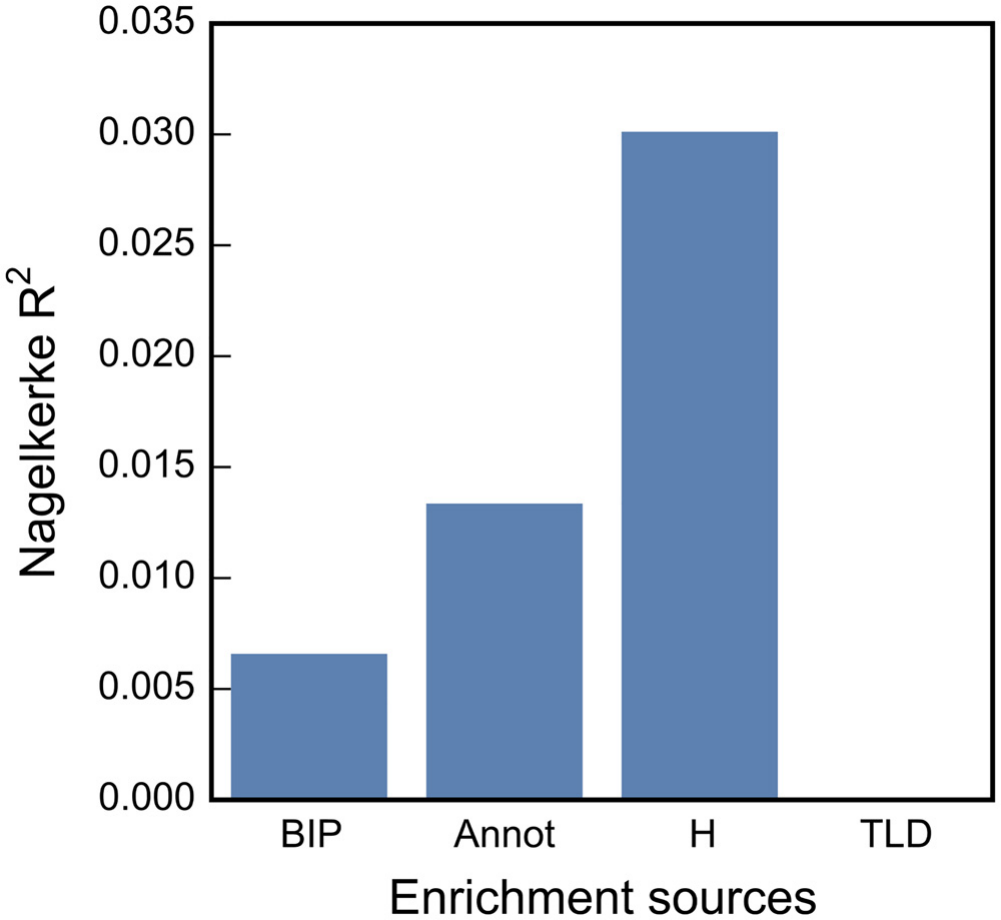
Relative importance of sources for enrichment. The relative importance of different sources of enrichment (x axis) for explaining SNP association with schizophrenia was measured by the Nagelkerke’s R^2^. The enrichment sources were: total linkage disequilibrium (TLD); the squared z-scores of SNP association with bipolar disorder (BIP); the LD weighted genomic annotation scores (Annot); and the heterozygosity (H).

We next evaluated the relative importance of the different categories of auxiliary information using the same thresholded logistic regression framework that we employed for constructing relative enrichment strata. The models without LD-weighted annotation scores (Annot), TLD, H, and BIP were each in turn compared with the full model, i.e., including all four categories (Materials and Methods). Fig 5 shows the relative importance of each source measured by change in Nagelkerke’s R^2^ (see S15 Fig, measured by the area under the receiver operating characteristic curve (AUC)). Annot, H, and BIP make major contributions to the enrichment of SNP association with SCZ (all having p < 10^−16^, likelihood ratio test). We find that the contribution of TLD is reduced when including other sources of differential enrichment. We also observe the same qualitative results when varying the *pthresh* used in dichotomizing the nominal p-values (S10A Fig). The contribution of TLD increases when we instead regress on the unthresholded z^2^ on enrichment sources and using change in adjusted R^2^ (S10B Fig), though it still remains smaller than the change in variance from excluding Annot or H categories, and is similar in size to the change in R^2^ excluding BIP. Of note, the proportion of explained variance in the unthresholded z^2^ regression is much smaller compared to that of the thresholded logistic regression (S10B Fig).

### Effect of Genetic Architecture Application to Other Phenotypes

To investigate the effect of different genetic architectures on the performance of CM3 we also analyzed the data for the brain structure Putamen volume[14] (26 sub-studies, N = 12,596) and Crohn’s diseases[15] (8 sub-studies, effective sample size N = 10,050). We identified 11 independent regions with predicted replication probability > 0.8 (S2 Table). Similarly, the number for Crohn’s disease is 81 (S3 Table). S12–S14 Figs show the performance of CM3 on these two additional datasets.

## Discussion

We have presented a novel algorithm, called CM3, which provides more accurate estimates of predicted replication probabilities for each SNP in a GWAS. Sorting SNPs based on predicted finite-sample replication probabilities incorporating auxiliary information, rather than by nominal p-values, yields a larger number of SNPs for a given replication threshold. The improved performance was demonstrated by comparing the average number of independent loci for a given observed cumulative replication rate in a split-half random partitioning analysis (Fig 4). Sorting SNPs based on the predicted replication probability was found to dramatically increase the yield of GWAS consistently across observed cumulative replication rates, relative to nominal p-values alone. We observed a broad range of replication probabilities across enrichment strata, for a given nominal p-value. Taken together, these results further demonstrate that “all SNPs are not created equal” [5], and that by leveraging differential enrichment across SNPs, it may be possible to improve on standard GWAS methods.

We have previously shown that LD-weighted genomic annotations and pleiotropy can be used to enrich the association of SNPs with SCZ in GWAS [5, 8, 9, 16], and we here demonstrate additional increase in power due to heterozygosity. Further enrichment was observed by adding association with another psychiatric phenotype, bipolar disorder, in line with previous findings of overlapping gene loci (pleiotropy)[8]. SNPs with higher heterozygosity replicate at higher rates, which is in accordance with the concept that common variants explain a large portion of the variance in complex human phenotypes[17] (S7 Fig). We used a multiple logistic regression model to investigate the relative contribution to the association with SCZ by the different sources of auxiliary information. By accounting for the overlap of contributions from different sources in this model, we found that total LD, LD-weighted genomic annotations, pleiotropy and heterozygosity contribute substantially to the association. We find that the contribution of total LD is substantially reduced when including these other sources of differential enrichment. The current analyses suggest that total LD partially acts as a proxy for other predictors of differential power and enrichment for non-null effects.

The current work is motivated by a previous paper from our group on LD-weighted enrichment annotation factors that appear to be related to very many complex traits and diseases [5]. The CM3 algorithm is based on a novel random partitioning approach to non-parametrically estimate replication effect size means and variances of GWAS summary statistics [18], along with a scale-mixture of two Gaussians modeling framework, similar to that proposed by Zhou *et al*.[19]. Unlike other partition-based approaches [20], the stratified CM3 model enables prediction of the posterior effect size and replication probability for each SNP, incorporating relative enrichment scores. Scale mixture models have been widely employed in genetic analyses, such as in animal genetic, GWAS, and QTL analysis[21]. The model used here differs from others in that: 1) it uses only summary statistics from GWAS sub-studies; 2) the estimation algorithm fits the model to the mean replication effect sizes and variances[18], allowing for estimation of the effects of changing effective sample sizes on the model fit; 3) the incorporation of enrichment scores obtained from SNP-level auxiliary information.

Several other methods which incorporating enrichment factors into GWAS have lately been developed [22–25]. The CM3 method differs from these previous methods in that: 1) none of the previously published methods directly model the proportion of null (the small-effect component) vs. non-null (large-effect component) effect sizes as a function of annotations; 2) none of the previously reported methods directly model the apparent inflation of the null distribution, which we do through allowing the null component to consist of very small replicating effects; 3) unlike previous methods, the current methodology produces estimates of replication effect sizes and replication probabilities from the random partitioning algorithm; 4) finally, we use the empirical predicted replication probabilities of SNPs as evidence of association since we can directly compute the empirical replication rate from the resampling experiment and directly compare this with the prediction performance of the corresponding CM3 covariate-stratified mixture of scale normal models.

It is of note that over-fitting could be a serious issue if the logistic regression was fitted selecting from a large number of annotation factors, especially trait/disease specific enrichment factors. This could be prevented by applying model selection procedures that guard against including too many predictors in the regression model. It is also possible that over-fitting can be an issue due to computing relative enrichment scores from a logistic regression model fitted using the entire dataset, though we validated the results by increased out-of-sample yield in SNPs for a given predicted replication rate (Fig 4B). Choice of number of strata to use for enrichment analyses and constructing smoothed “lookup tables” from the resulting fits is also an issue, which could potentially also be addressed by measures of model fit vs. model complexity trade-off. Note, though including auxiliary information (such as pleiotropic enrichment) in the CM3 will change the relative ranking of SNPs, the replication effect size estimates will not be affected negatively, e.g., SNPs related to BIP may have larger effect size estimates but SNPs unrelated to BIP will have unchanged effect size estimates.

The proposed method requires the summary statistics from multiple independent studies. The performance not only depends on the overall sample sizes and on the sizes of discovery and replication samples but also on the genetic architecture of the complex diseases/traits. In general, it performs better when the discovery sample is smaller comparing to the replication sample given the overall sample size is fixed (S6 and S14 Figs). This also suggests that CM3 may be more powerful for under-power studies. The results of applying CM3 to the data for the brain structure Putamen volume (putamen) and Crohn’s disease (CD) show that even with 8 independent sub-studies (CD) the improvement can be large. However, with 26 sub-studies available for Putamen volume the improvement is minimal (S12–S14 Figs).

An important utility of the CM3 method may be selection of a greater proportion of relevant SNPs for gene set enrichment and biological pathway analyses, which often use a less stringent p-value threshold than the established GWAS standard for discovery. Further, the proposed method may improve the efficiency of the two-stage GWAS meta-analysis by better predicting which regions will reach significance in the combined sample, relative to the standard method of picking all SNPs that reach a significance of p<1x10^-6^ in Stage I [26, 27].

In conclusion, we have presented a novel statistical method, the covariate modulated mixture model (CM3), which incorporates multiple sources of auxiliary information, such as total LD, heterozygosity, genomic annotations, and pleiotropy, for estimating effect sizes and predicting replication rates for SNPs in independent samples. The CM3 method first creates enrichment strata via multiple logistic regression, subsequently implementing a novel resampling-based algorithm to estimate replication effect sizes and probabilities non-parametrically. We then fit parametric models (scale mixtures of two normals) that minimize the sum of squared differences with the stratified nonparametric estimates; we show that these scale mixtures of normal provide good fits the stratified nonparametric estimates of effect size and replication probabilities for SCZ. The CM3 method does not depend on strong prior assumptions about the distribution of effect sizes, and the assumption of a scale mixture of two normal could be generalized to scale mixtures of three or more, to capture excess variation in the tails. By incorporating annotations, we show that the CM3 method results in larger numbers of identified SNPs (sensitivity) relative to the standard approach, when keeping replication rate (specificity) constant. The CM3 model may be further improved by incorporating more relevant prior information, such as gene expression, methylation, transcription regulation[10], chromatin marker annotation[28] and data about shared gene loci with other complex diseases, such as neurological disorders[8], cardiovascular disease factors[9] and immune-related diseases[29].

## Materials and Methods

### Data and Quality Control

The PGC SCZ data includes 35,476 cases and 46,839 controls[11]. Briefly, genotypes were filtered according to standard quality control parameters including: SNP missingness < 0.05, subject missingness < 0.02, and a test for deviation from Hardy-Weinberg equilibrium (P < 1x 10^−6^ in controls and 1x10^-10^ in cases). Related individuals were detected by using PLINK[30] with $\hat{\pi }\;>0.2$ with one individual from each pair removed. Principal components (PCs) were estimated using 39,239 SNPs with the program EIGENSOFT[31]. Genotype data were imputed using IMPUTE2[32] and SHAPEIT[33] based on the 1000 Genomes Project dataset. Association tests were performed on allele dosage data using the functions in PLINK with 11 PCs and study site indicators as covariates. Summary statistic p-values were generated by meta-analysis using an inverse-weighted fixed-effects model[34]. For detailed information, see the primary paper[11].

The bipolar disorder (BIP) sample consisting of the “BOMA-Bipolar”, the “Trinity College Dublin”, the “University of Edinburgh”, the “GlaxoSmithKline”, the “Systematic Treatment Enhancement Program for Bipolar Disorder”, the “University College London”, the “Thematically Organized Psychoses”, the “Wellcome Trust Case Control Consortium, WTCCC” and the “Research Program, Washington University at St. Louis, University of Pennsylvania, University of Chicago, Rush Medical School, University of Iowa, University of California, San Diego, University of California, San Francisco, and University of Michigan” studies from Sklar, *et al*.[7] were used in the current study. Genotype data were processed using the same QC parameters as for SCZ. Individuals related to or duplicated with the PGC SCZ sample were detected by PLINK with $\hat{\pi }\;>0.2$ and were removed. In total, 6,969 cases and 7,424 controls were analyzed. After QC procedures, sub-study data were combined and a mega-analysis was performed using PLINK, including the first 6 PCs and study site indicators as covariates.

### LD-informed Annotation Scores

A set of eight real-valued annotation scores, for each of the 9,266,541 SNPs analyzed here, were calculated based on the degree of correlation of the SNP with the eight different annotation categories that gave the highest genomic enrichment, as described in Schork *et al[5]*. These categories are: exon, intron, 5’ untranslated region (5’UTR), 3’ untranslated region (3’UTR), 1 and 10 kilo-basepairs upstream of the gene transcription start positions, and 1 and 10 kilo-basepairs downstream of gene transcription end positions in the UCSC database. Specifically, each score was computed as the sum of LD r^2^ for the given SNP with, respectively, SNPs in each of the eight positional categories, with these latter SNPs comprising the full set of SNPs in the 1000 Genomes Project (approximately 39 million, the European reference sample of the November 2012 release). SNPs were assigned to non-mutually exclusive annotation categories by thresholding the continuous category scores with an inclusive lower bound of 1.0; SNPs with scores below 1 on all functional categories were deemed *intergenic[5]*. In addition to annotation scores, the total LD (TLD) score for each SNP, given by the sum of all LD r^2^ for the SNP, was calculated. The correlation structure between pairs of categories is show in S1 Fig.

### Stratified Empirical Replication Effect Sizes

The 52 PGC SCZ sub-studies[11] were randomly partitioned 500 times. For each random partition, 26 of the PGC SCZ sub-studies were randomly assigned to the “discovery” sample and the complement to the “replication” sample. Inverse-variance based meta-analyses were then performed to calculate independent discovery and replication z-scores. Discovery z-scores were binned into 1,001 equally spaced intervals, and the average replication z-scores across all 500 iterations was computed for each bin. A cubic regression spline was fit to the ordinate axis (average replication z-scores) using the discovery z-score bin midpoints for the abscissa axis. This procedure was performed for all SNPs and also separately performed for strata defined by LD-weighted annotation categories (Fig 1A and S2 Fig), heterozygosity (Fig 1B), association levels with bipolar disorder (Fig 1C), and overall relative enrichment scores (described below; Fig 3A and S3 Fig).

### Relative Enrichment Score

Let *p*_*i*_ denote the p-value of the *i*th SNP from the full PGC sample. We define *Y*_*thresh*,*i*_ = 1 if *p*_*i*_≤*p*_*thresh*_ for a pre-set threshold *p*_*thresh*_ and *Y*_*thresh*,*i*_ = 0 otherwise. In the current study *p*_*thresh*_ = 10^−3^ was used (other choices of *p*_*thresh*_ lead to similar results, see S1 Text). A multiple logistic regression model was fit:*logit*[*p*_*r*_(*p*_*i*_≤*p*_*thresh*_|*X* = *x*_*i*_)] = *βx*_*i*_, where *x*_*i*_ are the values of the predictive variables for the *i*th SNP, i.e., annotation scores, total LD score, heterozygosity *H* = 2*k*(1-*k*) where *k* is the SNP minor allele frequency from the 1000 Genomes Project European subpopulation, and the squared z-score of the SNP association with bipolar disorder. The *relative enrichment score* for the *i*th SNP is defined as the estimated value of *P*_*r*_(*p*_*i*_≤*p*_*thresh*_|*X* = *x*_*i*_)from this model. Note, before computing the relative enrichment scores, SNPs located in the extended Major Histocompatibility Complex region (xMHC, chr6: 25652429–33368333, in total 6,467 SNPs) were removed and the remainder pruned at LD r^2^ < 0.8, i.e., keeping the SNP with the smallest p value in each LD block, so that in total 2,863,099 SNPs were analyzed.

### Gaussian Mixture Model

P-values from the GWAS studies were transformed into z-scores by the inverse standard normal cumulative distribution function, taking the same sign from the original study. The z-score of *i*th SNP can be modeled as $z_{i}=\sqrt{n}\delta_{i}+\varepsilon_{i}$, where *n* is the study effective sample size and *δ*_*1*_ is the effect size, independent of the zero-mean Gaussian residual error term $\varepsilon_{i}\sim N(0,\;\sigma_{0}^{2})$. In a commonly employed mixture model framework[13], it is assumed that some proportion *π*_*0*_ of SNP are null (*δ*_*1*_ = 0), and the proportion *π*_*1*_ = 1-*π*_*0*_ are non-null (*δ*1 ≠ 0) [13]. More generally, we make the assumption that an effect is “small” with prior probability *π*_*0*_ and “large” with prior probability *π*_*1*_. The class of “small effects” includes the possibility of null effects as a special case. The small effect component is modeled by the Gaussian density $\phi (0,\;nH_{i}\sigma^{2}{}_{1})$, and the large effect component is modeled by $\phi (0,\;nH_{i}(\sigma_{1}^{2}+\sigma_{2}^{2}))$, where *H*_*i*_ = 2*k*_*i*_(1-*k*_*i*_) is the heterozygosity and *k*_*i*_ is the minor allele frequency of the *i*th SNP. The two component densities for z-scores are thus

$$f_{0}(z_{i})=\phi (z_{i}|0,\;\sigma_{0}^{2}+nH_{i}\sigma_{1}^{2})$$

and

$$f_{1}(z_{i})=\phi (z_{i}|0,\;\sigma_{0}^{2}+nH_{i}(\sigma_{1}^{2}+\sigma_{2}^{2})).$$

The unconditional marginal mixture density for z-scores is then given by

$$f(z_{i})=\pi_{0}f_{0}(z_{i})+\pi_{1}f_{1}(z_{i})$$

Note, when $\sigma_{1}^{2}=0$, this reduces to the standard mixture of null (point mass at zero) and non-null (normally-distributed) z-scores.

### Posterior Effect Sizes and Predicted Replication Probabilities

Given the two component mixture model of effect sizes, the expected posterior effect size *δ*_*i*_ given *Z* = *z*_*i*_ is given by[13], p. 223,

$$\begin{array}{l} E\left\{\sqrt{n}\delta_{i}|Z=z_{i}\right\}=z_{i}+\sigma_{0}{}^{2}\frac{d}{dz_{i}}log\{f(z_{i})\}=z_{i}\left[Afdr(z_{i})+Btdr(z_{i})\right]\\ \;A=\;\frac{nH_{i}\sigma_{1}^{2}}{\sigma_{0}^{2}+nH_{i}\sigma_{1}^{2}}\;,\;B=\;\frac{nH_{i}(\sigma_{1}^{2}+\sigma_{2}^{2})}{\sigma_{0}^{2}+nH_{i}(\sigma_{1}^{2}+\sigma_{2}^{2})}\;, \end{array}$$ (1)

where *fdr*(*z*_*i*_) is the *local false discovery rate*, i.e., the posterior probability of a SNP being in the small effect component,

$$fdr(z_{i})=\pi_{0}f_{0}(z_{i})/f(z_{i}).$$ (2)

and *tdr*(*z*_*i*_) = 1-*fdr*(*z*_*i*_) is the *true discovery rate*, i.e., the posterior probability that a given SNP belongs to the large effect component.

The finite-sample *predicted replication probability* for a SNP *i* given the observed z-score *Z* = *z*_*i*_, is defined as the probability that the SNP in a *de novo* replication sample, with effective sample size *n*_*r*_, will have a z-score having the same sign and a magnitude equal or above certain threshold,*z*_*α*_. Formally, repl(z)i   =   Φ(−zα|μr,0,    σ2r,0)fdr(zi)+Φ(−zα|μr,1,    σ2r,1)tdr(zi), where ϕ is the Gaussian cumulative distribution function, and

$$\begin{array}{l} \mu_{r,0}=-\left(\frac{\sqrt{nn_{r}}H_{i}\sigma_{1}^{2}}{\sigma_{0}^{2}+nH_{i}\sigma_{1}^{2}}\right)\left|z_{i}\right|\\ \sigma_{r,0}^{2}=\sigma_{0}^{2}+n_{r}{}_{i}H_{i}\sigma_{1}^{2}-\frac{nn_{r}{\left(H_{i}\sigma_{1}^{2}\right)}^{2}}{\sigma_{0}^{2}+nH_{i}\sigma_{1}^{2}\;}\;\\ \mu_{r,1}=-\left(\frac{\sqrt{nn_{r}}H_{i}(\sigma_{1}^{2}+\sigma_{2}^{2})}{\sigma_{0}^{2}+nH_{i}(\sigma_{1}^{2}+\sigma_{2}^{2})}\right)\left|z_{i}\right|\\ \sigma_{r,2}^{2}=\sigma_{0}^{2}+n_{r}H_{i}(\sigma_{1}^{2}+\sigma_{2}^{2})-\frac{nn_{r}{\left(H_{i}(\sigma_{1}^{2}+\sigma_{2}^{2})\right)}^{2}}{\sigma_{0}^{2}+nH_{i}(\sigma_{1}^{2}+\sigma_{2}^{2})}\;. \end{array}$$ (3)

### Parameter Estimation

To estimate the model parameters, empirical replication effect sizes were calculated as described above, but in addition to the split-half discovery/replication breakdown of the 52 PGC sub-studies the procedure was repeated for discovery samples equal to 20%, 30%, and 40% of the total, with the complement being the replication sample. The four unknown parameters, $\pi_{1},\;\sigma_{0}^{2},\;\sigma_{1}^{2}$ and $\sigma_{2}^{2}$ from the scale-mixture of Gaussians model are then estimated by minimizing the squared differences between the model-based and empirical (nonparametric) estimates for the posterior mean effect size and the mean of the square of the effect size.

Note, each iteration of the procedure produces an unbiased estimate of the posterior effect size means and variances, conditional on the discovery z-scores. The purpose of averaging across 500 random iterations is to smooth out the random differences present in each arbitrary partition of the sample into discovery and replication samples. Since each iteration of the sample is unbiased, the average across all iterations is again unbiased for the conditional posterior means and variances. Details of the random partitioning procedure to produce the nonparametric estimates and the quadratic estimating equations used to estimate the mixture model parameters are detailed in the S1 Text.

To incorporate relative enrichment scores, SNPs are first stratified by predicted enrichment score computed from the logistic regression as described above. Nonparametric replication means and variances are computed for each stratum using the random partitioning procedure. Then, quadratic estimating equations are used to produce mixture model parameter estimates for each enrichment stratum separately. The predicted *a posteriori* effect sizes and replication probabilities are computed by Eqs (1) and (3), using the stratified parameter estimates from the quadratic estimating equations.

SNPs in the xMHC region were excluded, and the remaining 9,202,374 SNPs were randomly pruned using the LD structure from 1000 Genomes Project European subpopulation at r^2^ <0.8 (see S1 Text).

### Stratified Empirical Replication Probability

Discovery and replication z-scores were computed as described above from 500 random partitions. The–log_10_ p-values computed from the discovery z-scores were binned into 1001 equally spaced bins, and the proportion of SNPs in each bin with replication p-value < 0.05 was recoded as the *empirical replication rate*. The same procedure was performed on each stratum defined by predicted relative enrichment score (Fig 3B and S4 Fig). SNPs located in the xMHC region were removed and the remainder randomly pruned at LD r^2^ < 0.8 before performing the analysis (see S1 Text for random-pruning procedure).

### Relative Importance of Enrichment Sources

The sources of enrichment were grouped into four categories: LD-weighted genomic annotations, heterozygosity, pleiotropy with bipolar disorder (“pleiotropy” in this context is that the distribution of the summary statistics for one trait depends on those of another (“pleiotropic”) trait. No assumptions are made regarding the specific molecular, biological, or etiological factors underlying this relationship), and total LD score. Note, Then, four reduced logistic regression models were fitted. For each reduced model, one of the enrichment categories was excluded from the model, and the contribution of the deleted category was assessed by the difference in Nagelkerke’s R^2^ between the reduced model and the full model including all four categories. To investigate the effect of the threshold *p*_*thresh*_ used in dichotomizing the nominal p-values, this procedure was repeated with *p*_*thresh*_ = 10^−2^,10^−4^,10^−5^. As before, SNPs located in the xMHC region were removed and then pruned at LD r^2^ < 0.8, keeping the SNP with the smallest p-value in each LD block.

### Numerical Computation

See S1 Text for detailed numeric estimation of model parameters. Data QC and GWAS analysis were performed on the Genetic Cluster Computer hosted by the Dutch National Computing and Networking Services (http://www.geneticcluster.org/). And the polygenic analysis was performed using PLINK[30].

## Supporting Information

S1 Text Supporting Methods and author lists of PGC and ENIGMA. (PDF)

S1 Table Gene loci implicated by SNPs with predicted replication probability > 0.8 for Schizophrenia. The SNPs identified by CM3 at predicted replication probabilities > 0.8 were pruned at LD r^2^ < 0.1 (after removing SNPs in the xMHC region), keeping SNP having largest predicted replication probability in each LD block, and, clumped by 250kbp. Shaded rows indicate the genomic regions having SNP above genome wide significant threshold (5 x10^-8^). The locus number (Loci), leading SNPs (LeadingSNP), reference allele(A1), chromosome numbers (Chrnum), genomic position (Pos), predicted replication probability (Pred_Repl), p-values from the primary study (P), predicted replication probability excluding Bipolar disorder from the enrichment sources (Pred_Repl_noBIP) and closest genes in the region (Genes) are listed from left to right.
(XLS)

S2 Table Gene loci implicated by SNPs with predicted replication probability > 0.8 Crohn’s disease. The SNPs identified by CM3 at predicted replication probabilities > 0.8 were pruned at LD r^2^ < 0.1, keeping SNP having largest predicted replication probability in each LD block, and, clumped by 250kbp. Shaded rows indicate the genomic regions having SNP above genome wide significant threshold (5 x10^-8^). The locus number (Loci), leading SNPs (LeadingSNP), reference allele(A1), chromosome numbers (Chrnum), genomic position (Pos), predicted replication probability (Pred_Repl), p-values from the primary study (P), predicted replication probability excluding Bipolar disorder from the enrichment sources (Pred_Repl_noBIP) and closest genes in the region (Genes) are listed from left to right.
(XLS)

S3 Table Gene loci implicated by SNPs with predicted replication probability > 0.8 for Putamen volume. The SNPs identified by CM3 at predicted replication probabilities > 0.8 were pruned at LD r^2^ < 0.1 keeping SNP having largest predicted replication probability in each LD block, and, clumped by 250kbp. Shaded rows indicate the genomic regions having SNP above genome wide significant threshold (5 x10^-8^). The locus number (Loci), leading SNPs (LeadingSNP), reference allele(A1), chromosome numbers (Chrnum), genomic position (Pos), predicted replication probability (Pred_Repl), p-values from the primary study (P), predicted replication probability excluding Bipolar disorder from the enrichment sources (Pred_Repl_noBIP) and closest genes in the region (Genes) are listed from left to right.
(XLS)

S1 Fig Correlation between enrichment factors. Correlations between enrichment factors. The lower triangle shows Pearson’s correlation coefficient and upper triangle shows the Spearman’s rank correlation. The saturation of colour encodes the magnitude. The ellipses indicate the direction and magnitude. SNPs in the extended MHC region were removed and pruned based on the 1000 Genomes Project European population at r^2^ < 0.8, i.e. retain SNP having the smallest p-value from the full PGC SCZ sample in each LD block.
(EPS)

S2 Fig Stratified mean replication z-scores by all genomic categories studied. Mean replication z-scores for PGC SCZ SNPs from non-parametric estimates for categories stratified by LD-weighted annotation categories. The categories include exon, intron, 5’ un-translated region (5UTR), 3’ un-translated region (3UTR), 10 and 1 kilo-basepair upstream of the gene transcription start positions (10kup, 1kup), 10 and 1 kilo-basepair downstream of gene transcription end positions (10kdown, 1kdown) in the UCSC database, and, SNPs with scores below 1 on all functional categories (intergenic). 26 PGC SCZ sub-studies were randomly assigned as discovery sample and the remaining sub-studies as replication samples. All data were based on the average of 500 random draws.
(EPS)

S3 Fig Mean replication z-scores stratified by enrichment scores across different re-sampling proportions. The observed (solid lines) and predicted (dotted lines) mean z-scores in replication sample (y axis) were plotted against the z-scores in the discovery sample (x axis). The shrinkage of replication z-scores is differentiated by disjoint intervals of relative enrichment scores. **A**) 10, **B**) 17 and **C**) 20 PGC SCZ sub-studies were randomly assigned as discovery sample and the remaining sub-studies as replication samples. Colors indicate the 10 disjoint intervals or bins of relative enrichment scores, ranging from the least enriched stratum (Bin1) to the most enriched stratum (Bin10). All data were based on the average of 500 random draws. At each iteration the SNPs in the extended MHC region were removed and randomly pruned based on the 1000 Genomes Project European population at r^2^ < 0.8.
(EPS)

S4 Fig Mean replication probability stratified by enrichment scores across different re-sampling proportions. The observed (solid lines) and predicted (dotted lines) replication probabilities were plotted against the negative common logarithm of nominal p-values of Schizophrenia SNPs in discovery sample (x axis). **A**) 10, **B**) 17 and **C**) 20 PGC SCZ sub-studies were randomly assigned as discovery sample and the remaining sub-studies as replication samples. Colors indicate the 10 disjoint intervals or bins of relative enrichment scores, ranging from the least enriched (Bin1) to the most enriched stratum (Bin10). All data were based on the average of 500 random draws. At each iteration the SNPs in the extended MHC region were removed and randomly pruned based on the 1000 Genomes Project European population at r^2^ < 0.8.
(EPS)

S5 Fig Mean replication squared z-scores stratified by enrichment scores across different re-sampling proportions. The observed (solid lines) and predicted (dotted lines) squared mean z-scores in replication sample (y axis) were plotted against the z-scores in the discovery sample (x axis). **A**) 10, **B**) 17, **C**) 20 and **D**) 26 PGC sub-studies were randomly assigned as discovery sample and the remaining sub-studies as replication samples. Colors indicate the 10 disjoint intervals or bins of relative enrichment scores, ranging from the least enriched (Bin1) to the most enriched stratum (Bin10). All data were based on the average of 500 random draws. At each iteration the SNPs in the extended MHC region were removed and randomly pruned based on the 1000 Genomes Project European population at r^2^ < 0.8.
(EPS)

S6 Fig CM3 improves power of identifying gene loci by different splits. The average empirical cumulative replication rates (y axis) are plotted against the number of SNPs replicating at that rate > 0.5 (x axis), after removing MHC region SNPs and pruning at LD r^2^ < 0.1. Colors indicate different sorting criteria (green: sorted by prediction replication probability and blue sorted by nominal p values). **A**. For each iteration, 11 PGC schizophrenia sub-studies were randomly assigned to the discovery sample, and the rest to the replication sample. **B.** For each iteration, 16 PGC schizophrenia sub-studies were randomly assigned to the discovery sample, and the rest to the replication sample. **C.** For each iteration, 21 PGC schizophrenia sub-studies were randomly assigned to the discovery sample, and the rest to the replication sample. The average values over 500 iterations are shown.
(EPS)

S7 Fig Squared z-score as function of Heterozygosity. Linear relationship between Heterozygosity (H) and z^2^ statistic. A) uncorrected; B) corrected for imputation R^2^. The heterozygosity computed from the 1000 Genomes Project European population is divided into 500 equally spaced bins (x axis). Then, the mean z^2^ corresponding to each bin is plotted on the y-axis. The z^2^ and the imputation R^2^ for SNPs are obtained from the full PGC SCZ sample. SNPs in the extended MHC region were removed and pruned based on the 1000 Genomes Project European population at r^2^ < 0.8. Dotted line indicates the predicted confidence interval.
(EPS)

S8 Fig Ranks of SNP association with schizophrenia by CM3. The common logarithm of the rank of SNPs based on the predicted replication probability from CM3 (y axis), using the full PGC SCZ sample, is plotted against the ranks based on p-values (x axis). The change due to sorting by CM3 is most pronounced for SNPs having smaller effect sizes. The SNPs along the red dashed line indicate no change in the ranks. The full PGC SCZ sample was used and SNPs in the extended MHC region were removed and then pruned based on the 1000 Genomes Project European population at r^2^ < 0.8.
(EPS)

S9 Fig Mean replication z-score and replication rate for un-stratified data. **A.)** The observed (solid lines) and predicted (dotted lines) mean z-scores in replication sample (y axis) were plotted against the z-scores in the discovery sample (x axis). **B.)** The observed (solid lines) and predicted (dotted lines) replication probabilities were plotted against the negative common logarithm of nominal p values of SNPs in discovery sample (x axis). All data were generated by randomly assigning 26 of the PGC SCZ sub-studies as discovery sample and 26 as replication sample. The averaged value over 500 iterations was shown. At each iteration the SNPs in the extended MHC region were removed and randomly pruned based on the 1000 Genomes Project European population at r^2^ < 0.8. The GWAS significant threshold p = 5x10^-8^ (-log_10_(p) = 7.3) corresponding to a predicted replication rate 0.8571.
(EPS)

S10 Fig Relative contribution of enrichment sources. Explained variance by different enrichment sources. **A**) The–log_10_p SCZ is transformed to binary variable by different threshold (coded by colors) and the Nagelkerke’s R^2^ is computer by subtracting from the R^2^ of the full model the R^2^ of the reduced model, namely, excluding the corresponding source. **B**) The z^2^ SCZ is regressed on different enrichment sources. The adjusted R^2^ is computed by subtracting from the R^2^ of the full model the R^2^ of the reduced model. The full PGC SCZ sample was used and SNPs in the extended MHC region were removed and pruned based on the 1000 Genomes Project European population at r^2^ < 0.8.
(EPS)

S11 Fig Comparison between the CM3 and fGWAS methods by empirical replication rates. The average empirical cumulative replication rates (y axis) are plotted against the number of SNPs replicating at that rate > 0.5 (x axis), after removing MHC region SNPs and pruning at LD r^2^ < 0.1. The 52 PGC schizophrenia sub-studies were randomly split into discovery and replication groups 10 times, each with 26 sub-studies. The CM3 and fGWAS methods were applied to the discovery sample, including 10k up, 1kup, 5’UTR, exon, intron, 3’UTR, 1k down and 10k down as enrichment factors. In addition, the z-squared of the bipolar sample, heterozygosity and total LD were also included for CM3. The SNPs in the replication sample were sorted by the predicted replication probability (pred repl prob, green) and by posterior probability of association (PPA, blue) from fGWAS. The average empirical cumulative replication rates for the top 10,000 SNPs were plotted.
(EPS)

S12 Fig Mean replication z-scores stratified by enrichment scores across different re-sampling proportions for Crohn’s disease and Putamen volume. The observed (solid lines) and predicted (dotted lines) mean z-scores in replication sample (y axis) were plotted against the z-scores in the discovery sample (x axis). The shrinkage of replication z-scores is differentiated by disjoint intervals of relative enrichment scores. For Crohn’s disease: A) 2, B) 3 and C) 4 sub-studies out of 8 sub-studies were randomly assigned as discovery sample and the remaining sub-studies as replication samples. Colors indicate the 6 disjoint intervals or bins of relative enrichment scores, ranging from the least enriched stratum (Bin1) to the most enriched stratum (Bin6). All data were based on the average of all possible combination of random draws. Data was genomic inflation corrected before analysis. For Putamen volume, A) 8, B) 10 and C) 13 sub-studies out of 26 sub-studies were randomly assigned as discovery sample and the remaining sub-studies as replication samples. Colors indicate the 6 disjoint intervals or bins of relative enrichment scores, ranging from the least enriched stratum (Bin1) to the most enriched stratum (Bin6). All data were based on the average of 100 random draws. At each iteration, the SNPs were randomly pruned based on the 1000 Genomes Project European population at r^2^ < 0.8.
(EPS)

S13 Fig Mean replication probability stratified by enrichment scores across different re-sampling proportions for Crohn’s disease and Putamen volume. The observed (solid lines) and predicted (dotted lines) replication probabilities were plotted against the negative common logarithm of nominal p-values of SNPs in discovery sample (x axis). For Crohn’s disease: A) 2, B) 3 and C) 4 sub-studies out of 8 sub-studies were randomly assigned as discovery sample and the remaining sub-studies as replication samples. Colors indicate the 6 disjoint intervals or bins of relative enrichment scores, ranging from the least enriched stratum (Bin1) to the most enriched stratum (Bin6). All data were based on the average of all possible combination of random draws. Data was genomic inflation corrected before analysis. For Putamen volume, A) 8, B) 10 and C) 13 sub-studies out of 26 sub-studies were randomly assigned as discovery sample and the remaining sub-studies as replication samples. Colors indicate the 6 disjoint intervals or bins of relative enrichment scores, ranging from the least enriched stratum (Bin1) to the most enriched stratum (Bin6). All data were based on the average of 100 random draws. At each iteration the SNPs were randomly pruned based on the 1000 Genomes Project European population at r^2^ < 0.8.
(EPS)

S14 Fig CM3 improves power of identifying gene loci by different splits for Crohn’s disease and Putamen volume. The average empirical cumulative replication rates (y axis) are plotted against the number of SNPs replicating at that rate > 0.5 (x axis), after pruning at LD r^2^ < 0.1. Colors indicate different sorting criteria (green: sorted by prediction replication probability and blue sorted by nominal p values). For Crohn’s disease, at each iteration A. 2, B. 3 and C. 4 sub-studies out of 8 sub-studies were randomly assigned to the discovery sample, and the rest to the replication sample. The averaged values of all possible combination were shown. For Putamen volume, at each iteration, A. 8, B. 10 C.13 sub-studies out of 26 sub-studies were randomly assigned to the discovery sample, and the rest to the replication sample. The average values over 100 iterations are shown.
(EPS)

S15 Fig Relative importance of sources for enrichment measured by AUC. The relative importance of different sources of enrichment (x axis) for explaining SNP association with schizophrenia was measured by the improvement in the areas under the receiver operating characteristic curve (improvement in AUC). The enrichment sources were: total linkage disequilibrium (TLD); the squared z-scores of SNP association with bipolar disorder (BIP); the LD weighted genomic annotation scores (Annot); and the heterozygosity (H).
(EPS)
