# Supplementary material for: Leveraging Genomic Annotations and Pleiotropic Enrichment for Improved Replication Rates in Schizophrenia GWAS
Source: PLoS Genet. 2016 Jan 25;12(1):e1005803. doi: 10.1371/journal.pgen.1005803 (PMC4726519; doi:10.1371/journal.pgen.1005803)
Supplement: S1 Text — (PDF) [file pgen.1005803.s001.pdf]

Supporting Information

Text S1 Supporting Methods

Defining edges of enrichment strata

The predicted relative enrichment scores, $\hat{Y}$, we computed as the fitted value of $\log it(Y) = \beta X$ where *Y* is the probability that a SNP has p value smaller than a certain threshold, *pthresh*, and the *X* matrix contains enrichment categories, using the *glm* function with logistic link from the R statistical program. In order to make the stratified Q-Q plots equally spaced (horizontally) at the *pthresh=3* level, we deployed follow procedures. First, we applied the $\log_{10}$ transformation to $\hat{Y}$. Then we obtain $\tilde{Y}$ by applying a regression spline algorithm, using the $\hat{Y}$ on the ordinate axis and the Y on the absissa. This spline is then evaluated at 1,000 quantiles of $\hat{Y}$. We found the bin edges, for the 10 enrichment strata, of the conditional Q-Q by using the regression spline algorithm with $\tilde{Y}$ on the ordinate axis and $\hat{Y}$ on the absissa. We then applied the spline function on the 10 quantiles of $\tilde{Y}$.

Random Pruning

We performed random pruning before generating the empirical replication effect sizes for each iteration in the resampling procedure. Specifically, *N,* number of SNPs*,* random numbers were generated from the uniform distribution (0, 1). Then, using the LD matrix from the 1000 Genomes Project European population, only the data with maximum value in each LD block, defined by $r^2 < 0.8$, was marked. Finally, SNPs corresponding to the marked data point were retained and the rest were removed.

Parameter estimation of mixture model

With the two components scale mixture model, the expected posterior effect size δ_i given $Z_i = z_i$ are given by,

$$E\left\{\sqrt{n}\delta_i \mid Z = z_i\right\} = z_i + \sigma_0^2 \frac{d}{dz_i}\log\{f(z_i)\} = z_i\left[Afdr(z_i) + Btdr(z_i)\right]$$

$$A = \frac{nH_i\sigma_1^2}{\sigma_0^2 + nH_i\sigma_1^2},\quad B = \frac{nH_i(\sigma_1^2+\sigma_2^2)}{\sigma_0^2 + nH_i(\sigma_1^2+\sigma_2^2)} \qquad (1)$$

It is straightforward to show the posterior variance is given by

$$\begin{aligned} Var(\delta_i \mid Z = z_i) &= \sigma_0^2\left[1 + \sigma_0^2\frac{d^2}{dz^2}\log\{f(z_i)\}\right] \\ &= \sigma_0^2 + \sigma_0^4\left[\frac{z_i^2 - (\sigma_0^2 + nV_i\sigma_1^2)}{(\sigma_0^2 + nV_i\sigma_1^2)^2}fdr(z_i) + \right. \\ &\quad \frac{z_i^2 - (\sigma_0^2 + nV_i(\sigma_1^2+\sigma_2^2))}{(\sigma_0^2 + nV_i(\sigma_1^2+\sigma_2^2))^2}tdr(z_i) - \\ &\quad \left. z_i^2\left(\frac{fdr(z_i)}{\sigma_0^2 + nV_i\sigma_1^2} + \frac{tdr(z_i)}{\sigma_0^2 + nV_i(\sigma_1^2+\sigma_2^2)}\right)^2\right] \end{aligned} \qquad (2)$$

where n is the effective sample size, σ_0^2 the error variance, $f(z_i)$ the density of observed z-score, z_i, V_i the genotypic variance, $fdr(z_i)$ the local false discovery rate, $tdr(z_i)$ the true discovery rate for SNP *i*.

To estimate model parameters in the mixture model, $\sigma_0^2, \sigma_1^2, \sigma_2^2$ and π_1, we divided the PGC SCZ sub-studies into two disjoint groups. One group was assigned as the discovery and the other as replication samples. The number of sub-studies assigned as discovery sample was varied in increments of 10% from 20-50% (10, 17, 20 and 26 sub-studies) of all PGC sub-studies, and, for each scenario the complement sub-studies were used as replication sample. SNPs in the MHC region were first removed then pruned at LD $r^2 < 0.1$ before parameter estimation. Each scenario was iterated 500 times, with random selection of sub-studies and random pruning at each iteration. For each iteration *k*, *k=1,2, …, K(=500)*, we computed the meta-analysis discovery ($Z_{i[k]}$) and replication ($Z_{r,i[k]}$) Z-scores, *i=1, 2, …, N,* SNPs, using the fixed effect size formula[18], independently for discovery and replication samples. Then $Z_{i[k]}$ were binned into intervals $\mathrm{I}_m = [-10 + (m-1)h, -10 + mh]$ of width *h* on the interval [-10, 10), where *m=1,2, …, M* for *M=1,001*. Let $\aleph_{m[k]} = \{Z_{i[k]} : Z_{i[k]} \in \mathrm{I}_m\}$, and let $n_{m[k]} = card\{\aleph_{m[k]}\}$. We computed the sample means

$$\bar{Z}_{r,m[k]} = (1/n_{m[k]})\sum_{i:Z_{i[k]}\in\aleph_{m[k]}} Z_{r,i[k]} \text{ , (3)}$$

which are the nonparametric (empirical) estimates of $E\{Z_{r,m[k]} \mid Z_{m[k]}\}$. Similarly,

$$\bar{V}_{r,m[k]} = (1/n_{m[k]})\sum_{i:Z_{i[k]}\in\aleph_{m[k]}} Z^2_{r,i[k]} - \bar{Z}^2_{r,m[k]} \text{ (4)}$$

are the nonparametric estimates of the variance $Var\{Z_{r,m[k]} \mid Z_{m[k]}\}$. By entering equation (1) and (2) into the quadratic estimating equations, the parameters were estimated by minimizing the differences between the empirical (equation (3) and (4)) and model predicted expectation and variances (equation (1) and (2)).

Gene discovery

Finally, we used all PGC SCZ sample as discovery and assume there were a replication sample with the same size to compute the predicted replication probability for each SNPs.

Procedure of CM3 computation

$S = \{s_i \mid i=1,2, \ldots, m\}$, set of sub-studies available (m=52)
$\mathcal{M} = \{m_k \mid k=1,2, \ldots, k\}$, set of enrichment strata (k=10)
$\mathcal{F} = \{f \mid f \in (0.2, 0.3, 0.4, 0.5)\}$
$\mathcal{N} = \{n_j \mid j=1,2, \ldots, J\}$, set of iterations for random sampling for a specific split fraction, f
H, average heterozygosity of SNPs based on allele frequencies from the 1000 Genomes Project

Predicted enrichment score
Meta-analysis of all sub-studies
Pruning SNPs at LD $r^2 > 0.8$, i.e., SNPs with pair-wise $r^2 < 0.8$ are assumed to be independent and the SNP with smallest p value in a LD block was kept.
Computing predicted enrichment score using enrichment factors.

Stratifying SNPs
Dividing predicted enrichment score into k evenly distributed intervals
Assigning SNPs into the corresponding interval based on the predicted enrichment scores

Construct empirical discovery/replication dataset
for $n \in \mathcal{N}$
for $f \in \mathcal{F}$
compute the number of discovery sub-studies as discovery sample, $n_d = round(m * f)$
Randomly select n_d sub-studies, $S_{n,d}$, as discovery sample
The complementary sub-studies, $S_{n,r} = S \backslash S_{n,d}$, as replication sample
Compute meta-analysis z score for discovery sample and replication sample $z_{n,d}$, and $z_{n,r}$, respectively
Compute the effect sample size of $S_{n,d}$ and $S_{n,r}$, $N_{n,d}$ and $N_{n,r}$, respectively
Randomly prune SNPs using discovery sample, LD $r^2 < 0.8$
for $m \in \mathcal{M}$
Compute the observed condition replication z score for stratum m_k, $z_{n,f,m} = E(z_{n,r,m} / z_{n,d,m}, m)$

Predict posterior effect size
for $m \in \mathcal{M}$
Estimate $\pi_{1,m}, \sigma_{0,m}, \sigma_{1,m}, \sigma_{2,m}$ for stratum m based on H, $z_{\ldots,m}$, $z_{.,d,m}$, $N_{.,d}$ and $N_{.,r}$
i.e., the set of empirical data across all J iterations of resampling and all $\mathcal{F}$ split fractions
for $n \in \mathcal{N}$
Predict posterior effect size, $z_{p,n,d,m}$ based on $z_{n,d}$, $\pi_{1,m}, \sigma_{0,m}, \sigma_{1,m}, \sigma_{2,m}$, $N_{n,d}$ and $N_{n,r}$
Predict replication probability, $repl_{p,n,d,m}$ based on $z_{n,d}$, $\pi_{1,m}, \sigma_{0,m}, \sigma_{1,m}, \sigma_{2,m}$, $N_{n,d}$ and $N_{n,r}$
Compute average predicted posterior effect size $z_m = E_N(z_{p,n,d,m})$, i.e., averaged over J iterations
Compute average predicted posterior effect size $repl_m = E_N(repl_{p,n,d,m})$, i.e., averaged over J iterations

Predicted replication probability
Construct smooth 2-D lookup table of predicted replication probability for all SNPs based on the estimated parameter values (assuming there exists an independent sample with effective sample size equal to the current sample size, and, using the meta-analysis z score of all PGC2 sub-studies as discovery z scores)

Empirical replication rate
for $f \in \mathcal{F}$
for $n \in \mathcal{N}$
Number of discovery sub-studies as discovery sample, $n_d = round(m * f)$
Randomly sample n_d sub-studies, $S_{n,d}$, as discovery sample
The complementary sub-studies, $S_{n,r} = S \backslash S_{n,d}$, as replication sample
Compute meta-analysis z scores and p values for discovery sample and replication sample, $z_{n,d}$, $p_{n,d}$ and $z_{n,r}, p_{n,r}$ respectively.
Compute predicted replication probability, $repl_{n,d}$, based on estimated parameters $\pi_{1,m}, \sigma_{0,m}, \sigma_{1,m}, \sigma_{2,m}$, for $m \in \mathcal{M}$, and $z_{n,d}$
Prune SNPs using discovery sample, LD $r^2 < 0.1$, i.e., SNPs having pair-wise LD $r^2 < 0.1$ are assumed to be independent and keep the SNP with the highest replication rate or lowest $p_{n,d}$ in each LD block
Sort $p_{n,r}$ by $repl_{n,d}$, descendingly and by $p_{n,d}$ increasingly, separately
Compute empirical replication rates as the proportion SNPs with $p_{n,r,x} <= 0.05$ in the top X SNPs
Compute the average empirical replication rate for split f over J iterations

Bipolar Disorder and Schizophrenia Working Group of the Psychiatric Genomics Consortium

Stephan Ripke[1,2], Benjamin M. Neale[1,2,3,4], Aiden Corvin[5], James T. R. Walters[6], Kai-How Farh[1], Peter A. Holmans[6,7], Phil Lee[1,2,4], Brendan Bulik-Sullivan[1,2], David A. Collier[8,9], Hailiang Huang[1,3], Tune H. Pers[3,10,11], Ingrid Agartz[12,13,14], Esben Agerbo[15,16,17], Margot Albus[18], Madeline Alexander[19], Farooq Amin[20,21], Silviu A. Bacanu[22], Martin Begemann[23], Richard A Belliveau Jr[2], Judit Bene[24,25], Sarah E. Bergen [2,26], Elizabeth Bevilacqua[2], Tim B Bigdeli [22], Donald W. Black[27], Richard Bruggeman[28], Nancy G. Buccola[29], Randy L. Buckner[30,31,32], William Byerley[33], Wiepke Cahn[34], Guiqing Cai[35,36], Murray J. Cairns[39,119,168], Dominique Campion[37], Rita M. Cantor[38], Vaughan J. Carr[39,40], Noa Carrera[6], Stanley V. Catts[39,41], Kimberly D. Chambert[2], Raymond C. K. Chan[42], Ronald Y. L. Chen[43], Eric Y. H. Chen[43,44], Wei Cheng[45], Eric F. C. Cheung[46], Siow Ann Chong[47], C. Robert Cloninger[48], David Cohen[49], Nadine Cohen[50], Paul Cormican[5], Nick Craddock[6,7], Benedicto Crespo-Facorro[204], James J. Crowley[51], David Curtis[52,53], Michael Davidson[54], Kenneth L. Davis[36], Franziska Degenhardt[55,56], Jurgen Del Favero[57], Lynn E. DeLisi[127,128], Ditte Demontis[17,58,59], Dimitris Dikeos[60], Timothy Dinan[61], Gary Donohoe[5,62], Elodie Drapeau[36], Jubao Duan[63,64], Frank Dudbridge[65], Naser Durmishi[66], Peter Eichhammer[67], Johan Eriksson[68,69,70], Valentina Escott-Price[6], Laurent Essioux[71], Ayman H. Fanous[72,73,74,75], Martilias S. Farrell[51], Josef Frank[76], Lude Franke[77], Robert Freedman[78], Nelson B. Freimer[79], Marion Friedl[80], Joseph I. Friedman[36], Menachem Fromer[1,2,4,81], Giulio Genovese[2], Lyudmila Georgieva[6], Elliot S. Gershon[203], Ina Giegling[80,82], Paola Giusti-Rodríguez[51], Stephanie Godard[83], Jacqueline I. Goldstein[1,3], Vera Golimbet[84], Srihari Gopal[85], Jacob Gratten[86], Lieuwe de Haan[87], Christian Hammer[23], Marian L. Hamshere[6], Mark Hansen[88], Thomas Hansen[17,89], Vahram Haroutunian[36,90,91], Annette M. Hartmann[80], Frans A. Henskens[39,92,93], Stefan Herms[55,56,94], Joel N. Hirschhorn[3,11,95], Per Hoffmann[55,56,94], Andrea Hofman[55,56], Mads V. Hollegaard[96], David M. Hougaard[96], Masashi Ikeda[97], Inge Joa[98], Antonio Julià[99], René S. Kahn[34], Luba Kalaydjieva[100,101], Sena Karachanak-Yankova[102], Juha Karjalainen[77], David Kavanagh[6], Matthew C. Keller[103], Brian J. Kelly[119], James L. Kennedy[104,105,106], Andrey Khrunin[107], Yunjung Kim[51], Janis Klovins[108], James A. Knowles[109], Bettina Konte[80], Vaidutis Kucinskas[110], Zita Ausrele Kucinskiene[110], Hana Kuzelova-Ptackova[111], Anna K. Kähler[26], Claudine Laurent[19,112], Jimmy Lee Chee Keong[47,113], S. Hong Lee[86], Sophie E. Legge[6], Bernard Lerer[114], Miaoxin Li[43,44,115] Tao Li[116], Kung-Yee Liang[117], Jeffrey Lieberman[118], Svetlana Limborska[107], Carmel M. Loughland[39,119], Jan Lubinski[120], Jouko Lönnqvist[121], Milan Macek Jr[111], Patrik K. E. Magnusson[26], Brion S. Maher[122], Wolfgang Maier[123], Jacques Mallet[124], Sara Marsal[99], Manuel Mattheisen[17,58,59,125], Morten Mattingsdal[14,126], Robert W. McCarley[127,128], Colm McDonald[129], Andrew M. McIntosh[130,131], Sandra Meier[76], Carin J. Meijer[87], Bela Melegh[24,25], Ingrid Melle[14,132], Raquelle I. Mesholam-Gately[127,133], Andres Metspalu[134], Patricia T. Michie[39,135], Lili Milani[134], Vihra Milanova[136], Younes Mokrab[8], Derek W. Morris[5,62], Ole Mors[17,58,137], Kieran C. Murphy[138], Robin M. Murray[139], Inez Myin-Germeys[140], Bertram Müller-Myhsok[141,142,143], Mari Nelis[134], Igor Nenadic[144], Deborah A. Nertney[145], Gerald Nestadt[146], Kristin K. Nicodemus[147], Liene Nikitina-Zake[108], Laura Nisenbaum[148], Annelie Nordin[149], Eadbhard O'Callaghan[150], Colm O'Dushlaine[2], F. Anthony O'Neill[151], Sang-Yun Oh[152], Ann Olincy[78], Line Olsen[17,89], Jim Van Os[140,153], Christos Pantelis[39,154], George N. Papadimitriou[60], Sergi Papiol[23], Elena Parkhomenko[36], Michele T. Pato[109], Tiina Paunio[155,156], Milica Pejovic-Milovancevic[157], Diana O. Perkins[158], Olli Pietiläinen[156,159], Jonathan Pimm[53], Andrew J. Pocklington[6], John Powell[139], Alkes Price[3,160], Ann E. Pulver[146], Shaun M. Purcell[81], Digby Quested[161], Henrik B. Rasmussen[17,89], Abraham Reichenberg[36], Mark A. Reimers[162], Alexander L. Richards[6], Joshua L. Roffman[30,32], Panos Roussos[81,163], Douglas M. Ruderfer[6,81], Veikko Salomaa[70], Alan R. Sanders[63,64], Ulrich Schall[39,119], Christian R. Schubert[164], Thomas G. Schulze[76,165], Sibylle G. Schwab[166], Edward M. Scolnick[2], Rodney J. Scott[39,167,168], Larry J. Seidman[127,133], Jianxin Shi[169], Engilbert Sigurdsson[170], Hannes Petursson[170], Teimuraz Silagadze[171], Jeremy M. Silverman[36,172], Kang Sim[47], Petr Slominsky[107], Jordan W. Smoller[2,4], Hon-Cheong So[43], Chris C. A. Spencer[173], Eli A. Stahl[3,81], Hreinn Stefansson[174], Stacy Steinberg[174], Elisabeth Stogmann[175], Richard E. Straub[176], Eric Strengman[177,34], Jana Strohmaier[76],

T. Scott Stroup[118], Mythily Subramaniam[47], Jaana Suvisaari[121], Dragan M. Svrakic[48], Jin P. Szatkiewicz[51], Erik Söderman[12], Srinivas Thirumalai[178], Draga Toncheva[102], Paul A. Tooney[39,119,168], Sarah Tosato[179], Juha Veijola[180,181], John Waddington[182], Dermot Walsh[183], Dai Wang[85], Qiang Wang[116], Bradley T. Webb[22], Mark Weiser[54], Dieter B. Wildenauer[184], Nigel M. Williams[6], Stephanie Williams[51], Stephanie H. Witt[76], Aaron R. Wolen[162], Emily H. M. Wong[43], Brandon K. Wormley[22], Jing Qin Wu[39,168], Hualin Simon Xi[185], Clement C. Zai[104,105], Xuebin Zheng[186], Fritz Zimprich[175], Kari Stefansson[174], Rolf Adolfsson[149], Elvira Bramon[187], Joseph D. Buxbaum[35,36,90,188], Anders D. Børglum[17,58,59,137], Ariel Darvasi[189], Enrico Domenici[190], Hannelore Ehrenreich[23], Tõnu Esko[3,11,95,134], Pablo V. Gejman[63,64], Michael Gill[5], Hugh Gurling[53], Christina M. Hultman[26], Nakao Iwata[97], Assen V. Jablensky[39,101,184,191], Erik G. Jönsson[12,14], Kenneth S. Kendler[192], George Kirov[6], Jo Knight[104,105,106], Todd Lencz[193,194,195], Douglas F. Levinson[19], Qingqin S. Li[85], Jianjun Liu[186,196], Anil K. Malhotra[193,194,195], Steven A. McCarroll[2,95], Andrew McQuillin[53], Jennifer L. Moran[2], Preben B. Mortensen[15,16,17], Bryan J. Mowry[86,197], Roel A. Ophoff[38,79,34], Michael J. Owen[6,7], Aarno Palotie[2,4,159], Carlos N. Pato[109], Tracey L. Petryshen[2,127,198], Danielle Posthuma[199,200,201], Brien P. Riley[192], Dan Rujescu[80,82], Pak C. Sham[43,44,115], Pamela Sklar[81,90,163], David St Clair[202], Jens R. Wendland[164], Mark J. Daly[1,2,3], Patrick F. Sullivan[26,51,158,211], Laura J. Scott[205], Howard J. Edenberg[206,208], John I. Nurnberger[207,208], Douglas H. R. Blackwood[131,209,210], Matthew Flickinger[205], Weihua Guan[205], Phoenix Kwan[205], Thomas F. Wienker[212], Danyu Lin[213], Margit Burmeister[214], Tiffany A. Greenwood[215], Pierandrea Muglia[216], Erin N. Smith[217], Peter P. Zandi[218], Caroline M. Nievergelt[215], Rebecca Mckinney[215], Paul D. Shilling[215], Nicholas J. Schork[219], Cinnamon S. Bloss[217], Tatiana Foroud[207], Daniel L. Koller[207], Chunyu Liu[220], Judith A. Badner[220], William A. Scheftner[221], William B. Lawson[222], Evaristus A. Nwulia[222], Maria Hipolito[222], William Coryell[223], John P. Rice[224], Francis J. McMahon[225], Wade Berrettini[226], Falk W. Lohoff[226], James B. Potash[218], Pamela B. Mahon[218], Melvin G. Mcinnis[227], Sebastian Zöllner[227], Peng Zhang[227], David W. Craig[228], Szabocls Szelinger[228], Thomas B. Barrett[229], René Breuer[76], Federica Tozzi[216], Anne Farmer[9], Peter McGuffin[9], John Strauss[230], Wei Xu[231], John B. Vincent[230], Keith Matthews[232], Richard Day[233], Manuel D.C. Ferreira[2,234,235], Roy Perlis[2,234], Soumya Raychaudhuri[2,234], Phil L. Hyoun[234], Jun Li[214], Devin Absher[236], Robert C. Thompson[237], Fan Guo Meng[238], Kevin Mcghee[209,210], Alan F. Schatzberg[19], William E. Bunney[237], Jack D. Barchas[238], Edward G. Jones[239], Stanley J. Watson[240], Richard M. Myers[236], Huda Akil[240], Michael Boehnke[205], Kim Chambert[2], Ed Scolnick[2], Gunnar Morken[241,242], Emma Quinn[5,81], Thomas W. Mühleisen[55,56], Johannes Schumacher[56], Michael Steffens[212], Peter Propping[56], Adebayo Anjorin[211], Nick Bass[211], Radhika Kandaswamy[211], Jacob Lawrence[211], Alan W. Mclean[209,210], Walter J. Muir[209,210], Benjamin S. Pickard[209,210], Gerome Breen[9,202], Sian Caesar[243], Katherine Gordon-Smith[6,243], Lisa Jones[243], Christine Fraser[6], Elaine K. Green[6], Detelina Grozeva[6], Ian R. Jones[6], Valentina Moskvina[6,216], Ivan Nikolov[6,81], Amanda Elkin[9], Richard Williamson[9], Allan H. Young[232,244], I Nicol Ferrier[232], Porgeir Porgeirsson[174], Omar Gustafsson[174], Vishwajit Nimgaonkar[245], Mikael Landén[26,246], Paul Lichtenstein[26], Martin Schalling[247], Urban Osby[247], Lena Backlund[12], Louise Frisén[247], Niklas Langstrom[246], Stéphane Jamain[248,249,250], Marion Leboyer[248,249,250], Bruno Etain[248,249,250], Frank Bellivier[248,249,250], Susanne Lucae[142], Markus Schwarz[251], Peter R. Schofield[252,253], Nick Martin[235], Grant W. Montgomery[235], Mark Lathrop[254], Högni Oskarsson[255], Michael Bauer[256], Adam Wright[257], Philip B. Mitchell[257], Martin Hautzinger[258], Andreas Reif[259] & John R. Kelsoe,[215,260]

[1]Analytic and Translational Genetics Unit, Massachusetts General Hospital, Boston, Massachusetts 02114, USA.

[2]Stanley Center for Psychiatric Research, Broad Institute of MIT and Harvard, Cambridge, Massachusetts 02142, USA.

[3]Medical and Population Genetics Program, Broad Institute of MIT and Harvard, Cambridge, Massachusetts 02142, USA.

[4]Psychiatric and Neurodevelopmental Genetics Unit, Massachusetts General Hospital, Boston, Massachusetts 02114, USA.

[5]Neuropsychiatric Genetics Research Group, Department of Psychiatry, Trinity College Dublin, Dublin 8, Ireland.

[6]MRC Centre for Neuropsychiatric Genetics and Genomics, Institute of Psychological Medicine and Clinical Neurosciences, School of Medicine, Cardiff University, Cardiff, CF24 4HQ, UK.

[7]National Centre for Mental Health, Cardiff University, Cardiff, CF24 4HQ, UK.

[8]Eli Lilly and Company Limited, Erl Wood Manor, Sunninghill Road, Windlesham, Surrey, GU20 6PH, UK.

[9]Social, Genetic and Developmental Psychiatry Centre, Institute of Psychiatry, King's College London, London, SE5 8AF, UK.

[10]Center for Biological Sequence Analysis, Department of Systems Biology, Technical University of Denmark, DK-2800, Denmark.

[11]Division of Endocrinology and Center for Basic and Translational Obesity Research, Boston Children's Hospital, Boston, Massachusetts 02115, USA.

[12]Department of Clinical Neuroscience, Psychiatry Section, Karolinska Institutet, Stockholm, SE-17176, Sweden.

[13]Department of Psychiatry, Diakonhjemmet Hospital, Oslo 0319, Norway.

[14]NORMENT, KG Jebsen Centre for Psychosis Research, Institute of Clinical Medicine, University of Oslo, Oslo 0424, Norway.

[15]Centre for Integrative Register-based Research, CIRRAU, Aarhus University, Aarhus DK-8210, Denmark.

[16]National Centre for Register-based Research, Aarhus University, Aarhus DK-8210, Denmark.

[17]The Lundbeck Foundation Initiative for Integrative Psychiatric Research, iPSYCH, Denmark.

[18]State Mental Hospital, Haar 85540, Germany.

[19]Department of Psychiatry and Behavioral Sciences, Stanford University, Stanford, California 94305, USA.

[20]Department of Psychiatry and Behavioral Sciences, Atlanta Veterans Affairs Medical Center, Atlanta, Georgia 30033, USA.

[21]Department of Psychiatry and Behavioral Sciences, Emory University, Atlanta Georgia 30322, USA.

[22]Virginia Institute for Psychiatric and Behavioral Genetics, Department of Psychiatry, Virginia Commonwealth University, Richmond, Virginia 23298, USA.

[23]Clinical Neuroscience, Max Planck Institute of Experimental Medicine, Göttingen 37075, Germany.

[24]Department of Medical Genetics, University of Pécs, Pécs H-7624, Hungary.

[25]Szentagothai Research Center, University of Pécs, Pécs H-7624, Hungary.

[26]Department of Medical Epidemiology and Biostatistics, Karolinska Institutet, Stockholm SE-17177, Sweden.

[27]Department of Psychiatry, University of Iowa Carver College of Medicine, Iowa City, Iowa 52242, USA.

[28]University Medical Center Groningen, Department of Psychiatry, University of Groningen, Groningen NL-9700 RB, The Netherlands.

[29]School of Nursing, Louisiana State University Health Sciences Center, New Orleans, Louisiana 70112, USA.

[30]Athinoula A. Martinos Center, Massachusetts General Hospital, Boston, Massachusetts 02129, USA.

[31]Center for Brain Science, Harvard University, Cambridge, Massachusetts, 02138 USA.

[32]Department of Psychiatry, Massachusetts General Hospital, Boston, Massachusetts 02114 USA.

[33]Department of Psychiatry, University of California at San Francisco, San Francisco, California 94143 USA.

[34]University Medical Center Utrecht, Department of Psychiatry, Rudolf Magnus Institute of Neuroscience, Utrecht 3584, The Netherlands.

[35]Department of Human Genetics, Icahn School of Medicine at Mount Sinai, New York, New York 10029 USA.

[36]Department of Psychiatry, Icahn School of Medicine at Mount Sinai, New York, New York 10029 USA.

[37]Centre Hospitalier du Rouvray and INSERM U1079 Faculty of Medicine, Rouen 76301, France.

[38]Department of Human Genetics, David Geffen School of Medicine, University of California, Los Angeles, California 90095, USA.

[39]Schizophrenia Research Institute, Sydney NSW 2010, Australia.

[40]School of Psychiatry, University of New South Wales, Sydney NSW 2031, Australia.

[41]Royal Brisbane and Women's Hospital, University of Queensland, Brisbane, St Lucia QLD 4072, Australia.

[42]Institute of Psychology, Chinese Academy of Science, Beijing 100101, China.

[43]Department of Psychiatry, Li Ka Shing Faculty of Medicine, The University of Hong Kong, Hong Kong, China.

[44]State Key Laboratory for Brain and Cognitive Sciences, Li Ka Shing Faculty of Medicine, The University of Hong Kong, Hong Kong, China.

[45]Department of Computer Science, University of North Carolina, Chapel Hill, North Carolina 27514, USA.

[46]Castle Peak Hospital, Hong Kong, China.

[47]Institute of Mental Health, Singapore 539747, Singapore.

[48]Department of Psychiatry, Washington University, St. Louis, Missouri 63110, USA.

[49]Department of Child and Adolescent Psychiatry, Assistance Publique Hopitaux de Paris, Pierre and Marie Curie Faculty of Medicine and Institute for Intelligent Systems and Robotics, Paris 75013, France.

[50] Blue Note Biosciences, Princeton, New Jersey 08540, USA

[51]Department of Genetics, University of North Carolina, Chapel Hill, North Carolina 27599-7264, USA.

[52]Department of Psychological Medicine, Queen Mary University of London, London E1 1BB, UK.

[53]Molecular Psychiatry Laboratory, Division of Psychiatry, University College London, London WC1E 6JJ, UK.

[54]Sheba Medical Center, Tel Hashomer 52621, Israel.

[55]Department of Genomics, Life and Brain Center, Bonn D-53127, Germany.

[56]Institute of Human Genetics, University of Bonn, Bonn D-53127, Germany.

[57]Applied Molecular Genomics Unit, VIB Department of Molecular Genetics, University of Antwerp, Antwerp B-2610, Belgium.

[58]Centre for Integrative Sequencing, iSEQ, Aarhus University, Aarhus DK-8000 , Denmark.

[59]Department of Biomedicine, Aarhus University, Aarhus DK-8000, Denmark.

[60]First Department of Psychiatry, University of Athens Medical School, Athens 11528, Greece.

[61]Department of Psychiatry, University College Cork, Co. Cork, Ireland.

[62]Cognitive Genetics and Therapy Group, School of Psychology and Discipline of Biochemistry, National University of Ireland Galway, Co. Galway, Ireland.

[63]Department of Psychiatry and Behavioral Neuroscience, University of Chicago, Chicago, Illinois 60637, USA.

[64]Department of Psychiatry and Behavioral Sciences, NorthShore University HealthSystem, Evanston, Illinois 60201, USA.

[65]Department of Non-Communicable Disease Epidemiology, London School of Hygiene and Tropical Medicine, London WC1E 7HT, UK.

[66]Department of Child and Adolescent Psychiatry, University Clinic of Psychiatry, Skopje 1000, Republic of Macedonia.

[67]Department of Psychiatry, University of Regensburg, Regensburg 93053, Germany.

[68]Department of General Practice, Helsinki University Central Hospital, University of Helsinki P.O. Box 20, Tukholmankatu 8 B, Helsinki FI-00014, Finland

[69]Folkhälsan Research Center, Helsinki, Finland, Biomedicum Helsinki 1, Haartmaninkatu 8, Helsinki FI-00290, Finland.

[70]National Institute for Health and Welfare, P.O. BOX 30, Helsinki FI-00271, Finland.

[71]Translational Technologies and Bioinformatics, Pharma Research and Early Development, F. Hoffman-La Roche, Basel CH-4070, Switzerland.

[72]Department of Psychiatry, Georgetown University School of Medicine, Washington DC 20057, USA.

[73]Department of Psychiatry, Keck School of Medicine of the University of Southern California, Los Angeles, California 90033, USA.

[74]Department of Psychiatry, Virginia Commonwealth University School of Medicine, Richmond, Virginia 23298, USA.

[75]Mental Health Service Line, Washington VA Medical Center, Washington DC 20422, USA.

[76]Department of Genetic Epidemiology in Psychiatry, Central Institute of Mental Health, Medical Faculty Mannheim, University of Heidelberg, Heidelberg , Mannheim D-68159, Germany.

[77]Department of Genetics, University of Groningen, University Medical Centre Groningen, Groningen 9700 RB, The Netherlands.

[78]Department of Psychiatry, University of Colorado Denver, Aurora, Colorado 80045, USA.

[79]Center for Neurobehavioral Genetics, Semel Institute for Neuroscience and Human Behavior, University of California, Los Angeles, California 90095, USA.

[80]Department of Psychiatry, University of Halle, Halle 06112, Germany.

[81]Division of Psychiatric Genomics, Department of Psychiatry, Icahn School of Medicine at Mount Sinai, New York, New York 10029, USA.

[82]Department of Psychiatry, University of Munich, Munich 80336, Germany.

[83]Departments of Psychiatry and Human and Molecular Genetics, INSERM, Institut de Myologie, Hôpital de la Pitiè-Salpêtrière, Paris 75013, France.

[84]Mental Health Research Centre, Russian Academy of Medical Sciences, Moscow 115522, Russia.

[85]Neuroscience Therapeutic Area, Janssen Research and Development, Raritan, New Jersey 08869, USA.

[86]Queensland Brain Institute, The University of Queensland, Brisbane, Queensland, QLD 4072, Australia.

[87]Academic Medical Centre University of Amsterdam, Department of Psychiatry, Amsterdam 1105 AZ, The Netherlands.

[88]Illumina, La Jolla, California, California 92122, USA.

[89]Institute of Biological Psychiatry, Mental Health Centre Sct. Hans, Mental Health Services, Copenhagen DK-4000, Denmark.

[90]Friedman Brain Institute, Icahn School of Medicine at Mount Sinai, New York, New York 10029, USA.

[91]J. J. Peters VA Medical Center, Bronx, New York, New York 10468, USA.

[92]Priority Research Centre for Health Behaviour, University of Newcastle, Newcastle NSW 2308, Australia.

[93]School of Electrical Engineering and Computer Science, University of Newcastle, Newcastle NSW 2308, Australia.

[94]Division of Medical Genetics, Department of Biomedicine, University of Basel, Basel CH-4058, Switzerland.

[95]Department of Genetics, Harvard Medical School, Boston, Massachusetts 02115, USA.

[96]Section of Neonatal Screening and Hormones, Department of Clinical Biochemistry, Immunology and Genetics, Statens Serum Institut, Copenhagen DK-2300, Denmark.

[97]Department of Psychiatry, Fujita Health University School of Medicine, Toyoake, Aichi 470-1192, Japan.

[98]Regional Centre for Clinical Research in Psychosis, Department of Psychiatry, Stavanger University Hospital, Stavanger 4011, Norway.

[99]Rheumatology Research Group, Vall d'Hebron Research Institute, Barcelona 08035, Spain.

[100]Centre for Medical Research, The University of Western Australia, Perth WA 6009, Australia.

[101]The Perkins Institute for Medical Research, The University of Western Australia, Perth WA 6009, Australia.

[102]Department of Medical Genetics, Medical University, Sofia1431, Bulgaria.

[103]Department of Psychology, University of Colorado Boulder, Boulder, Colorado 80309, USA.

[104]Campbell Family Mental Health Research Institute, Centre for Addiction and Mental Health, Toronto, Ontario M5T 1R8, Canada.

[105]Department of Psychiatry, University of Toronto, Toronto, Ontario M5T 1R8, Canada.

[106]Institute of Medical Science, University of Toronto, Toronto, Ontario M5S 1A8, Canada.

[107]Institute of Molecular Genetics, Russian Academy of Sciences, Moscow123182, Russia.

[108]Latvian Biomedical Research and Study Centre, Riga LV-1067, Latvia.

[109]Department of Psychiatry and Zilkha Neurogenetics Institute, Keck School of Medicine at University of Southern California, Los Angeles, California 90089, USA.

[110]Faculty of Medicine, Vilnius University, LT-01513 Vilnius, Lithuania.

[111]Department of Biology and Medical Genetics, 2nd Faculty of Medicine and University Hospital Motol, Prague 150 06, Czech Republic.

[112] Department of Child and Adolescent Psychiatry, Pierre and Marie Curie Faculty of Medicine, Paris 75013, France.

[113]Duke-NUS Graduate Medical School, Singapore 169857, Singapore.

[114]Department of Psychiatry, Hadassah-Hebrew University Medical Center, Jerusalem 91120, Israel.

[115]Centre for Genomic Sciences, The University of Hong Kong, Hong Kong, China.

[116]Mental Health Centre and Psychiatric Laboratory, West China Hospital, Sichuan University, Chengdu, Sichuan 610041, China.

[117]Department of Biostatistics, Johns Hopkins University Bloomberg School of Public Health, Baltimore, Maryland 21205, USA.

[118]Department of Psychiatry, Columbia University, New York, New York 10032, USA.

[119]Priority Centre for Translational Neuroscience and Mental Health, University of Newcastle, Newcastle NSW 2300, Australia.

[120]Department of Genetics and Pathology, International Hereditary Cancer Center, Pomeranian Medical University in Szczecin, Szczecin 70-453, Poland.

[121]Department of Mental Health and Substance Abuse Services; National Institute for Health and Welfare, P.O. BOX 30, Helsinki FI-00271, Finland

[122]Department of Mental Health, Bloomberg School of Public Health, Johns Hopkins University, Baltimore, Maryland 21205, USA.

[123]Department of Psychiatry, University of Bonn, Bonn D-53127, Germany.

[124]Centre National de la Recherche Scientifique, Laboratoire de Génétique Moléculaire de la Neurotransmission et des Processus Neurodégénératifs, Hôpital de la Pitié Salpêtrière, Paris 75013, France.

[125]Department of Genomics Mathematics, University of Bonn, Bonn D-53127, Germany.

[126]Research Unit, Sørlandet Hospital, Kristiansand 4604, Norway.

[127]Department of Psychiatry, Harvard Medical School, Boston, Massachusetts 02115, USA.

[128]VA Boston Health Care System, Brockton, Massachusetts 02301, USA.

[129]Department of Psychiatry, National University of Ireland Galway, Co. Galway, Ireland.

[130]Centre for Cognitive Ageing and Cognitive Epidemiology, University of Edinburgh, Edinburgh EH16 4SB, UK.

[131]Division of Psychiatry, University of Edinburgh, Edinburgh EH16 4SB, UK.

[132]Division of Mental Health and Addiction, Oslo University Hospital, Oslo 0424, Norway.

[133]Massachusetts Mental Health Center Public Psychiatry Division of the Beth Israel Deaconess Medical Center, Boston, Massachusetts 02114, USA.

[134]Estonian Genome Center, University of Tartu, Tartu 50090, Estonia.

[135]School of Psychology, University of Newcastle, Newcastle NSW 2308, Australia.

[136]First Psychiatric Clinic, Medical University, Sofia 1431, Bulgaria.

[137]Department P, Aarhus University Hospital, Risskov DK-8240, Denmark.

[138]Department of Psychiatry, Royal College of Surgeons in Ireland, Dublin 2, Ireland.

[139]King's College London, London SE5 8AF, UK.

[140]Maastricht University Medical Centre, South Limburg Mental Health Research and Teaching Network, EURON, Maastricht 6229 HX, The Netherlands.

[141]Institute of Translational Medicine, University of Liverpool, Liverpool L69 3BX, UK.

[142]Max Planck Institute of Psychiatry, Munich 80336, Germany.

[143]Munich Cluster for Systems Neurology (SyNergy), Munich 80336, Germany.

[144]Department of Psychiatry and Psychotherapy, Jena University Hospital, Jena 07743, Germany.

[145]Department of Psychiatry, Queensland Brain Institute and Queensland Centre for Mental Health Research, University of Queensland, Brisbane, Queensland St Lucia QLD 4072, Australia.

[146]Department of Psychiatry and Behavioral Sciences, Johns Hopkins University School of Medicine, Baltimore, Maryland 21205, USA.

[147]Department of Psychiatry, Trinity College Dublin, Dublin 2, Ireland.

[148]Eli Lilly and Company, Lilly Corporate Center, Indianapolis, Indiana 46285, USA.

[149]Department of Clinical Sciences, Psychiatry, Umeå University, Umeå SE-901 87, Sweden.

[150]DETECT Early Intervention Service for Psychosis, Blackrock, Co. Dublin, Ireland.

[151]Centre for Public Health, Institute of Clinical Sciences, Queen's University Belfast, Belfast BT12 6AB, UK.

[152]Lawrence Berkeley National Laboratory, University of California at Berkeley, Berkeley, California 94720, USA.

[153]Institute of Psychiatry, King's College London, London SE5 8AF, UK.

[154]Melbourne Neuropsychiatry Centre, University of Melbourne & Melbourne Health, Melbourne, Vic 3053, Australia.

[155]Department of Psychiatry, University of Helsinki, P.O. Box 590, Helsinki FI-00029 HUS, Finland.

[156]Public Health Genomics Unit, National Institute for Health and Welfare, P.O. BOX 30, Helsinki FI-00271, Finland

[157]Medical Faculty, University of Belgrade, Belgrade 11000, Serbia.

[158]Department of Psychiatry, University of North Carolina, Chapel Hill, North Carolina 27599-7160, USA.

[159]Institute for Molecular Medicine Finland, FIMM, University of Helsinki, P.O. Box 20, Helsinki FI-00014, Finland

[160]Department of Epidemiology, Harvard School of Public Health, Boston, Massachusetts 02115, USA.

[161]Department of Psychiatry, University of Oxford, Oxford OX3 7JX, UK.

[162]Virginia Institute for Psychiatric and Behavioral Genetics, Virginia Commonwealth University, Richmond, Virginia 23298, USA.

[163]Institute for Multiscale Biology, Icahn School of Medicine at Mount Sinai, New York, New York 10029, USA.

[164]PharmaTherapeutics Clinical Research, Pfizer Worldwide Research and Development, Cambridge, Massachusetts 02139, USA.

[165]Department of Psychiatry and Psychotherapy, University of Gottingen, Göttingen 37073, Germany.

[166]Psychiatry and Psychotherapy Clinic, University of Erlangen, Erlangen 91054, Germany.

[167]Hunter New England Health Service, Newcastle NSW 2308, Australia.

[168]School of Biomedical Sciences and Pharmacy, University of Newcastle, Callaghan NSW 2308, Australia.

[169]Division of Cancer Epidemiology and Genetics, National Cancer Institute, Bethesda, Maryland 20892, USA.

[170]University of Iceland, Landspitali, National University Hospital, Reykjavik 101, Iceland.

[171]Department of Psychiatry and Drug Addiction, Tbilisi State Medical University (TSMU), **N33, 0177** Tbilisi, Georgia.

[172]Research and Development, Bronx Veterans Affairs Medical Center, New York, New York 10468, USA.

[173]Wellcome Trust Centre for Human Genetics, Oxford OX3 7BN, UK.

[174]deCODE Genetics, Reykjavik 101, Iceland.

[175]Department of Clinical Neurology, Medical University of Vienna, Wien 1090, Austria.

[176]Lieber Institute for Brain Development, Baltimore, Maryland 21205, USA.

[177]Department of Medical Genetics, University Medical Centre Utrecht, Universiteitsweg 100, Utrecht 3584 CG, The Netherlands.

[178]Berkshire Healthcare NHS Foundation Trust, Bracknell RG12 1BQ, UK.

[179]Section of Psychiatry, University of Verona, Verona 37134, Italy.

[180]Department of Psychiatry, University of Oulu, P.O. BOX 5000, Oulu FI-90014, Finland

[181]University Hospital of Oulu, P.O.BOX 20, 90029 OYS, Finland.

[182]Molecular and Cellular Therapeutics, Royal College of Surgeons in Ireland, Dublin 2, Ireland.

[183]Health Research Board, Dublin 2, Ireland.

[184]School of Psychiatry and Clinical Neurosciences, The University of Western Australia, Perth WA6009, Australia.

[185]Computational Sciences CoE, Pfizer Worldwide Research and Development, Cambridge, Massachusetts 02139, USA.

[186]Human Genetics, Genome Institute of Singapore, A*STAR, Singapore 138672, Singapore.

[187]University College London, London WC1E 6BT, UK.

[188]Department of Neuroscience, Icahn School of Medicine at Mount Sinai, New York, New York 10029, USA.

[189]Department of Genetics, The Hebrew University of Jerusalem, Jerusalem 91905, Israel.

[190]Neuroscience Discovery and Translational Area, Pharma Research and Early Development, F. Hoffman-La Roche, Basel CH-4070, Switzerland.

[191]Centre for Clinical Research in Neuropsychiatry, School of Psychiatry and Clinical Neurosciences, The University of Western Australia, Medical Research Foundation Building, Perth WA 6000, Australia.

[192]Virginia Institute for Psychiatric and Behavioral Genetics, Departments of Psychiatry and Human and Molecular Genetics, Virginia Commonwealth University, Richmond, Virginia 23298, USA.

[193]The Feinstein Institute for Medical Research, Manhasset, New York 11030 USA.

[194]The Hofstra NS-LIJ School of Medicine, Hempstead, New York 11549 USA.

[195]The Zucker Hillside Hospital, Glen Oaks, New York 11004 USA.

[196]Saw Swee Hock School of Public Health, National University of Singapore, Singapore 117597, Singapore.

[197]Queensland Centre for Mental Health Research, University of Queensland, Brisbane 4076, Queensland, Australia.

[198]Center for Human Genetic Research and Department of Psychiatry, Massachusetts General Hospital, Boston, Massachusetts 02114, USA.

[199]Department of Child and Adolescent Psychiatry, Erasmus University Medical Centre, Rotterdam 3000, The Netherlands.

[200]Department of Complex Trait Genetics, Neuroscience Campus Amsterdam, VU University Medical Center Amsterdam, Amsterdam 1081, The Netherlands.

[201]Department of Functional Genomics, Center for Neurogenomics and Cognitive Research, Neuroscience Campus Amsterdam, VU University, Amsterdam 1081, The Netherlands.

[202]University of Aberdeen, Institute of Medical Sciences, Aberdeen, AB25 2ZD, UK.

[203]Departments of Psychiatry and Human Genetics, University of Chicago, Chicago, Illinois 60637, USA.

[204]University Hospital Marqués de Valdecilla, Instituto de Formación e Investigación Marqués de Valdecilla, University of Cantabria, Santander E-39008, Spain

[205]Department of Biostatistics and Center for Statistical Genetics, School of public health, University of Michigan, Ann Arbor, Michigan 48109, USA

[206]Department of Biochemistry and Molecular Biology, Indiana University School of Medicine, Indianapolis, Indiana 46202, USA

[207]Department of Psychiatry, Indiana University School of Medicine, Indianapolis, Indiana 46202, USA

[208]Department of Medical and Molecular Genetics, Indiana University School of Medicine, Indianapolis, Indiana 46202, USA

[209]Division of Psychiatry, University of Edinburgh, Royal Edinburgh Hospital, Edinburgh EH10 5HF, UK

[210]Medical Genetics Section, University of Edinburgh, Western General Hospital, Molecular Medicine Centre, Edinburgh EH4 2XU, UK

[211]Medstar Research Institute, Baltimore, Maryland 20782, USA

[212]Institute of Medical Biometry, Informatics and Epidemiology, Bonn Universität - University of Bonn, Bonn D-53127, Germany

[213]Department of Biostatistics, University of North Carolina, Chapel Hill, North Carolina 27599, USA

[214]Department of Human Genetics, Department of Psychiatry, Molecular and Behavioral Neuroscience Institute, University of Michigan, Ann Arbor, Michigan 48109, USA

[215]Department of Psychiatry, University of California San Diego, La Jolla, California 92093, USA

[216]Neurosciences Centre of Excellence in Drug Discovery, GlaxoSmithKline Research and Development, Verona 37135, Italy

[217]The Scripps Translational Science Institute and Scripps Health, La Jolla, California 92037, USA

[218]Department of Mental Health, Johns Hopkins University and Hospital, Baltimore, Maryland 21205, USA

[219]The Scripps Translational Science Institute and The Scripps Research Institute, La Jolla, California 92037, USA

[220]Department of Psychiatry, University of Chicago, Chicago, Illinois 60637, USA

[221]Rush University Medical Center, Chicago, Illinois 60612, USA

[222]Department of Psychiatry and Behavioral Sciences, Howard University College of Medicine, Washington, DC 20001, USA

[223]Department of Psychiatry, University of Iowa, Iowa City 52242, USA

[224]Washington University School of Medicine, St Louis, Missouri 63110, USA

[225]National Institute of Mental Health NIH, Bethesda, Maryland 20892, USA

[226]Department of Psychiatry, University of Pennsylvania, Philadelphia, Pennsylvania 19104, USA

[227]Department of Psychiatry, University of Michigan, Ann Arbor, Michigan 48109, USA

[228]The Translational Genomics Research Institute, Phoenix, Arizona 85004, USA

[229]Portland Veterans Affairs Medical Center, Portland, Oregon 97239, USA

[230]Molecular Neuropsychiatry and Development Laboratory, Centre for Addiction and Mental Health, Toronto, Ontario M5S 2S1, Canada

[231]Department of Biostatistics, Princess Margaret Hospital, Toronto, Ontario M5G 2M9, CA

[232]School of Medicine, University of Dundee, Nethergate, Dundee DD1 9SY, UK

[233]School of Neurology, Neurobiology and Psychiatry Royal Victoria Infirmary, Newcastle upon Tyne NE1 4LP, UK

[234]Center for Human Genetic Research, Massachusetts General Hospital, Harvard Medical School, Boston, Massachusetts 02114, USA

[235]Queensland Institute of Medical Research, Brisbane, Qld 4029, Australia

[236]HudsonAlpha Institute for Biotechnology, Huntsville, Alabama 35806, USA

[237]Department of Psychiatry and Human Behavior, University of California, Irvine, California 92868, USA

[238]Department of Psychiatry, Weill Medical College, Cornell University, New York, New York 10065, USA

[239]Department of Psychiatry and Behavioral Sciences, Center for Neuroscience, University of California, Davis-Livermore, California 95817, USA

[240]Molecular and Behavioral Neuroscience Institute, University of Michigan, Ann Arbor, Michigan 48109, USA

[241]Department of Psychiatry, St Olavs Hospital, Trondheim 7006, Norway

[242]Department of Neuroscience, Norwegian University of Science and Technology, Trondheim 7489, Norway

[243]Department of Psychiatry, School of Clinical and Experimental Medicine, Birmingham University, Birmingham B15 2TT, UK

[244]Institute of Mental Health, University of British Columbia (UBC), Vancouver, British Columbia V6T 2A1, Canada

[245]Department of Human Genetics, University of Pittsburgh, Pittsburgh, Pennsylvania 15261, USA

[246]Insitute of Neuroscience and Physiology University of Gothenburg, University of Gothenburg, Sweden, Box 100, Gothenburg S-405 30, Sweden

[247]Department of Molecular Medicine, Karolinska Institutet, Stockholm SE-17176, Sweden

[248]Institut Mondor de Recherche Biomédicale INSERM : U955, Université Paris-Est Créteil Val-de-Marne (UPEC), IFR10, 8 rue du Général Sarrail, Créteil 94010, France

[249]Service de psychiatrie Assistance publique - Hôpitaux de Paris (AP-HP), Hôpital Henri Mondor, Hôpital Albert Chenevier, Créteil 94010, France

[250]ENBREC, European Network of Bipolar Research Expert Centres ENBREC, Créteil 94000, France

[251]Psychiatric Center Nordbaden, Wiesloch D-69168, Germany

[252]Prince of Wales Medical Institute, Sydney NSW 2031, Australia

[253]University of New south Wales, Sydney NSW 2052, Australia

[254]CNG, Centre National de Génotypage CEA : DSV/IG, Centre National de Génotypage 2 rue Gaston Crémieux CP5721 EVRY 91057, Cedex France

[255]Therapeia, Reykjavik IS-101, Iceland

[256]Department of Psychiatry and Psychotherapy, ENBREC Group, University Hospital Carl Gustav Carus, Dresden 01307, Germany

[257]School of Psychiatry, University of New South Wales, Black Dog Institute, Sydney, New South Wales 2031, Australia

[258]Department of Clinical and Developmental Psychology, University of Tubingen, Institute of Psychology, Tubingen 72072, Germany

[259]Department of Psychiatry University of Würzburg, Würzburg 97080, Germany

[260]Department of Psychiatry, Special Treatment and Evaluation Program (STEP) Veterans Affairs San Diego Healthcare System, San Diego, California 92093, USA

Enhancing Neuro Imaging Genetics through Meta Analysis (ENIGMA) Consortium participant list:

Derrek P. Hibar*[1], Jason L. Stein*[1,2], Miguel E. Renteria*[3], Alejandro Arias-Vasquez*[4,5,6,7], Sylvane Desrivières*[8], Neda Jahanshad[1], Roberto Toro[9], Katharina Wittfeld[10,11], Lucija Abramovic[12], Micael Andersson[13], Benjamin S. Aribisala[14,15,16], Nicola J. Armstrong[17,18], Manon Bernard[19], Marc M. Bohlken[12], Marco P. Boks[12], Janita Bralten[4,6,7], Andrew A. Brown[20,21], M. Mallar Chakravarty[22,23], Qiang Chen[24], Christopher R.K. Ching[1], Gabriel Cuellar-Partida[3], Anouk den Braber[25], Sudheer Giddaluru[26,27], Aaron L. Goldman[24], Oliver Grimm[28], Tulio Guadalupe[29,30], Johanna Hass[31], Girma Woldehawariat[32], Avram J. Holmes[33,34], Martine Hoogman[4,7], Deborah Janowitz[11], Tianye Jia[8], Sungeun Kim[35,36,37], Marieke Klein[4,7], Bernd Kraemer[38], Phil H. Lee[39,34,40,41], Loes M. Olde Loohuis[42], Michelle Luciano[43], Christine Macare[8], Karen A. Mather[17], Manuel Mattheisen[44,45,46], Yuri Milaneschi[47], Kwangsik Nho[35,36,37], Martina Papmeyer[48], Adaikalavan Ramasamy[49,50], Shannon L. Risacher[35,37], Roberto Roiz-Santiañez[51,52], Emma J. Rose[53], Alireza Salami[13], Philipp G. Sämann[54], Lianne Schmaal[47], Andrew J. Schork[55,56], Jean Shin[19], Lachlan T. Strike[3,57], Alexander Teumer[58], Marjolein M.J. van Donkelaar[4,7], Kristel R. van Eijk[12], Raymond K. Walters[59,60], Lars T. Westlye[21,61], Christopher D. Whelan[62], Anderson M. Winkler[63], Marcel P. Zwiers[7], Saud Alhusaini[64,62], Lavinia Athanasiu[20,21], Stefan Ehrlich[31,34,65], Marina M.H. Hakobjan[4,7], Cecilie B. Hartberg[20,66], Unn Haukvik[20,66], Angelien J.G.A.M. Heister[4,7], David Höhn[54], Dalia Kasperaviciute[67,68], David C.M. Liewald[43], Lorna M. Lopez[43], Remco R.R. Makkinje[4,7], Mar Matarin[67], Marlies A.M. Naber[4,7], David R. McKay[69,70], Margaret Needham[53], Allison C. Nugent[32], Benno Pütz[54], Natalie A. Royle[14,43,16], Li Shen[35,36,37], Emma Sprooten[48,69,70], Daniah Trabzuni[50,71], Saskia S.L. van der Marel[4,7], Kimm J.E. van Hulzen[4,7], Esther Walton[31], Christiane Wolf[54], Laura Almasy[72], David Ames[73,74], Sampath Arepalli[75], Amelia A. Assareh[17], Mark E. Bastin[14,43,76,16], Henry Brodaty[17,77], Kazima B. Bulayeva[78], Melanie A. Carless[72], Sven Cichon[79,80,81], Aiden Corvin[53], Joanne E. Curran[72], Michael Czisch[54], Greig I. de Zubicaray[57], Allissa Dillman[75], Ravi Duggirala[72], Thomas D. Dyer[72], Susanne Erk[82], Iryna O. Fedko[25], Luigi Ferrucci[83], Tatiana M. Foroud[84,37], Peter T. Fox[85], Masaki Fukunaga[86], Raphael Gibbs[75,50], Harald H.H. Göring[72], Robert C. Green[87,40], Sebastian Guelfi[50], Narelle K. Hansell[3], Catharina A. Hartman[88], Katrin Hegenscheid[89], Andreas Heinz[82], Dena G. Hernandez[75,50], Dirk J. Heslenfeld[90], Pieter J. Hoekstra[88], Florian Holsboer[54], Georg Homuth[91], Jouke-Jan Hottenga[25], Masashi Ikeda[92], Clifford R. Jack Jr.[93], Mark Jenkinson[94], Robert Johnson[95], Ryota Kanai[96,97], Maria Keil[38], Jack W. Kent Jr[72], Peter Kochunov[98], John B. Kwok[99,100], Stephen M. Lawrie[48], Xinmin Liu[32,101], Dan L. Longo[102], Katie L. McMahon[103], Eva Meisenzahl[104], Ingrid Melle[20,21], Sebastian Mohnke[82], Grant W. Montgomery[3], Jeanette C. Mostert[4,7], Thomas W. Mühleisen[81,80], Michael A. Nalls[75], Thomas E. Nichols[105,94], Lars G. Nilsson[13], Markus M. Nöthen[80,106], Kazutaka Ohi[107], Rene L. Olvera[85], Rocio Perez-Iglesias[108,52], G. Bruce Pike[109,110], Steven G. Potkin[111], Ivar Reinvang[61], Simone Reppermund[17], Marcella Rietschel[28], Nina Romanczuk-Seiferth[82], Glenn D. Rosen[112,113], Dan Rujescu[104], Knut Schnell[114], Peter R. Schofield[99,100], Colin Smith[115], Vidar M. Steen[26,27], Jessika E. Sussmann[48], Anbupalam Thalamuthu[17], Arthur W. Toga[116], Bryan Traynor[75], Juan Troncoso[117], Jessica A. Turner[118], Maria C. Valdés Hernández[76], Dennis van 't Ent[25], Marcel van der Brug[119], Nic J.A. van der Wee[120], Marie-Jose van Tol[121], Dick J. Veltman[47], Thomas H. Wassink[122], Eric Westman[123], Ronald H. Zielke[95], Alan Zonderman[124], David G. Ashbrook[125],

Reinmar Hager[125], Lu Lu[126,127], Francis J. McMahon[32], Derek W. Morris[128,53], Robert W. Williams[126,127], Han G. Brunner[4,7,129], Randy L. Buckner[130,34], Jan K. Buitelaar[6,7,131], Wiepke Cahn[12], Vince D. Calhoun[132,133], Gianpiero L. Cavalleri[62], Benedicto Crespo-Facorro[51,52], Anders M. Dale[134,135], Gareth E. Davies[136], Norman Delanty[137,62], Chantal Depondt[138], Srdjan Djurovic[20,139], Wayne C. Drevets[32,140], Thomas Espeseth[61,21], Randy L. Gollub[34,65,40], Beng-Choon Ho[141], Wolfgang Hoffmann[58,10], Norbert Hosten[89], René S. Kahn[12], Stephanie Le Hellard[26,27], Andreas Meyer-Lindenberg[28], Bertram Müller-Myhsok[54,142,143], Matthias Nauck[144], Lars Nyberg[13], Massimo Pandolfo[138], Brenda W.J.H. Penninx[47], Joshua L. Roffman[34], Sanjay M. Sisodiya[67], Jordan W. Smoller[39,34,40,41], Hans van Bokhoven[4,7], Neeltje E.M. van Haren[12], Henry Völzke[58], Henrik Walter[82], Michael W. Weiner[145], Wei Wen[17], Tonya White[146,147], Ingrid Agartz[20,66,148], Ole A. Andreassen[20,21], John Blangero[72], Dorret I. Boomsma[25], Rachel M. Brouwer[12], Dara M. Cannon[32,149], Mark R. Cookson[75], Eco J.C. de Geus[25], Ian J. Deary[43], Gary Donohoe[128,53], Guillén Fernández[6,7], Simon E. Fisher[29,7], Clyde Francks[29,7], David C. Glahn[69,70], Hans J. Grabe[11,150], Oliver Gruber[38,54], John Hardy[50], Ryota Hashimoto[151], Hilleke E. Hulshoff Pol[12], Erik G. Jönsson[148,20], Iwona Kloszewska[152], Simon Lovestone[153,154], Venkata S. Mattay[24], Patrizia Mecocci[155], Colm McDonald[149], Andrew M. McIntosh[48], Roel A. Ophoff[42,12], Tomas Paus[156,157], Zdenka Pausova[19,158], Mina Ryten[50,49], Perminder S. Sachdev[17,159], Andrew J. Saykin[35,37,84], Andy Simmons[160,161,162], Andrew Singleton[75], Hilkka Soininen[163,164], Joanna M. Wardlaw[14,43,76,16], Michael E. Weale[49], Daniel R. Weinberger[24,165], Hieab H.H. Adams[166,147], Lenore J. Launer[167], Stephan Seiler[168], Reinhold Schmidt[168], Ganesh Chauhan[169], Claudia L. Satizabal[170,171], James T. Becker[172,173,174], Lisa Yanek[175], Sven J. van der Lee[166], Maritza Ebling[65,176], Bruce Fischl[65,176], W.T. Longstreth[177], Douglas Greve[65,176], Helena Schmidt[178], Paul Nyquist[179], Louis N. Vinke[65,176], Cornelia M. van Duijn[166], Xue Luting[180], Bernard Mazoyer[181], Joshua C. Bis[182], Vilmundur Gudnason[183], Sudha Seshadri[170,171], M. Arfan Ikram[166,147], the Alzheimer's Disease Neuroimaging Initiative**, the CHARGE Consortium, EPIGEN, IMAGEN, SYS, Nicholas G. Martin*[3], Margaret J. Wright*[3,57], Gunter Schumann*[8], Barbara Franke*[4,5,7], Paul M. Thompson*[+1], Sarah E. Medland*[+3]

**Data used in preparing this article were obtained from the Alzheimer's Disease Neuroimaging Initiative (ADNI) database (adni.loni.usc.edu). As such, many investigators within the ADNI contributed to the design and implementation of ADNI and/or provided data but did not participate in analysis or writing of this report. A complete listing of ADNI investigators can be found at: http://adni.loni.usc.edu/wp-content/uploads/how_to_apply/ADNI_Acknowledgement_List.pdf
*Denotes equal contribution
[+]Denotes the corresponding authors

1. Imaging Genetics Center, Institute for Neuroimaging & Informatics, Keck School of Medicine of the University of Southern California, Los Angeles, USA
2. Neurogenetics Program, Department of Neurology, UCLA School of Medicine, Los Angeles, USA
3. QIMR Berghofer Medical Research Institute, Brisbane, Australia
4. Department of Human Genetics, Radboud University Medical Center, Nijmegen, The Netherlands
5. Department of Psychiatry, Radboud University Medical Center, Nijmegen, The Netherlands
6. Department of Cognitive Neuroscience, Radboud University Medical Center, Nijmegen, The Netherlands
7. Donders Institute for Brain, Cognition and Behaviour, Raboud University, Nijmegen, The Netherlands
8. MRC-SGDP Centre, Institute of Psychiatry, King's College, London, London, United Kingdom
9. Institut Pasteur, Paris, France

10. German Center for Neurodegenerative Diseases (DZNE), Rostock/Greifswald, Germany, Greifswald, Germany
11. Department of Psychiatry, University Medicine Greifswald, Greifswald, Germany
12. Brain Center Rudolf Magnus, Department of Psychiatry, UMC Utrecht, Utrecht, The Netherlands
13. Umeå Centre for Functional Brain Imaging (UFBI), Umeå University, Umeå, Sweden
14. Brain Research Imaging Centre, University of Edinburgh, Edinburgh, United Kingdom
15. Department of Computer Science, Lagos State University, Lagos, Nigeria
16. Scottish Imaging Network, A Platform for Scientific Excellence (SINAPSE) Collaboration, Department of Neuroimaging Sciences, University of Edinburgh, Edinburgh, United Kingdom
17. Centre for Healthy Brain Ageing, School of Psychiatry, University of New South Wales (UNSW), Sydney, Australia
18. School of Mathematics and Statistics, University of Sydney, Australia
19. Hospital for Sick Children, University of Toronto, Toronto, Canada
20. NORMENT - KG Jebsen Centre, Institute of Clinical Medicine, University of Oslo, Oslo, Norway
21. NORMENT - KG Jebsen Centre, Division of Mental Health and Addiction, Oslo University Hospital, Oslo, Norway
22. Cerebral Imaging Centre, Douglas Mental Health University Institute, Montreal, Canada
23. Department of Psychiatry and Biomedical Engineering, McGill University, Montreal, Canada
24. Lieber Institute for Brain Development, Baltimore, USA
25. Biological Psychology, Neuroscience Campus Amsterdam, VU University & VU Medical Center, Amsterdam, The Netherlands
26. NORMENT - KG Jebsen Centre for Psychosis Research, Department of Clinical Science, University of Bergen, Norway
27. Dr. Einar Martens Research Group for Biological Psychiatry, Center for Medical Genetics and Molecular Medicine, Haukeland University Hospital, Bergen, Norway
28. Central Institute of Mental Health, Medical Faculty Mannheim, University Heidelberg, Mannheim, Germany
29. Language and Genetics Department, Max Planck Institute for Psycholinguistics, Nijmegen, The Netherlands
30. International Max Planck Research School for Language Sciences, Nijmegen, The Netherlands
31. Department of Child and Adolescent Psychiatry, Faculty of Medicine of the TU Dresden, Dresden, Germany
32. National Institute of Mental Health Intramural Research Program, Bethesda, USA
33. Department of Psychology, Yale University, New Haven, USA
34. Department of Psychiatry, Massachusetts General Hospital, Boston, USA
35. Center for Neuroimaging, Radiology and Imaging Sciences, Indiana University School of Medicine, Indianapolis, USA
36. Center for Computational Biology and Bioinformatics, Indiana University School of Medicine, Indianapolis, USA
37. Indiana Alzheimer Disease Center, Indiana University School of Medicine, Indianapolis, USA
38. Center for Translational Research in Systems Neuroscience and Psychiatry, Department of Psychiatry and Psychotherapy, University Medical Center, Goettingen, Germany
39. Psychiatric and Neurodevelopmental Genetics Unit, Center for Human Genetic Research, Massachusetts General Hospital, Boston, USA
40. Harvard Medical School, Cambridge, USA

41. Stanley Center for Psychiatric Research, Broad Institute of MIT and Harvard, Boston, USA
42. Center for Neurobehavioral Genetics, University of California, Los Angeles, USA
43. Centre for Cognitive Ageing and Cognitive Epidemiology, Psychology, University of Edinburgh, Edinburgh, United Kingdom
44. Department of Biomedicine, Aarhus University, Aarhus, Denmark
45. The Lundbeck Foundation Initiative for Integrative Psychiatric Research, iPSYCH, Aarhus and Copenhagen, Denmark
46. Center for integrated Sequencing, iSEQ, Aarhus University, Aarhus, Denmark
47. Department of Psychiatry, Neuroscience Campus Amsterdam, VU University Medical Center, Amsterdam, The Netherlands
48. Division of Psychiatry, Royal Edinburgh Hospital, University of Edinburgh, Edinburgh, United Kingdom
49. Department of Medical and Molecular Genetics, King's College London, London, United Kingdom
50. Reta Lila Weston Institute and Department of Molecular Neuroscience, UCL Institute of Neurology, London, United Kingdom
51. Department of Psychiatry, University Hospital Marqués de Valdecilla, School of Medicine, University of Cantabria-IDIVAL, Santander, Spain
52. Cibersam (Centro Investigación Biomédica en Red Salud Mental), Madrid, Spain
53. Neuropsychiatric Genetics Research Group, Department of Psychiatry and Trinity College Institute of Psychiatry, Trinity College Dublin, Ireland
54. Max Planck Institute of Psychiatry, Munich, Germany
55. Multimodal Imaging Laboratory, Department of Neurosciences, University of California, San Diego, USA
56. Department of Cognitive Sciences, University of California, San Diego, USA
57. School of Psychology, University of Queensland, Brisbane, Australia
58. Institute for Community Medicine, University Medicine Greifswald, Greifswald, Germany
59. Analytic and Translational Genetics Unit, Massachusetts General Hospital, Boston, USA
60. Medical and Population Genetics Program, Broad Institute for Harvard and MIT, Cambridge, USA
61. NORMENT - KG Jebsen Centre, Department of Psychology, University of Oslo, Oslo, Norway
62. Molecular and Cellular Therapeutics, The Royal College of Surgeons, Dublin, Ireland
63. Oxford University, Oxford, United Kingdom
64. Department of Neurology and Neurosurgery, Montreal Neurological Institute, McGill University, Montreal, Canada
65. Martinos Center for Biomedical Imaging, Massachusetts General Hospital, Charlestown, USA
66. Department of Research and Development, Diakonhjemmet Hospital, Oslo, Norway
67. UCL Institute of Neurology, London, United Kingdom and Epilepsy Society, United Kingdom
68. Department of Medicine, Imperial College London, London, UK
69. Department of Psychiatry, Yale University, New Haven, USA
70. Olin Neuropsychiatric Research Center, Hartford, USA
71. Department of Genetics, King Faisal Specialist Hospital and Research Centre, Riyadh, Saudi Arabia
72. Texas Biomedical Research Institute, San Antonio, USA
73. National Ageing Research Institute, Royal Melbourne Hospital, Melbourne, Australia
74. Academic Unit for Psychiatry of Old Age, University of Melbourne, Australia
75. Laboratory of Neurogenetics, National Institute on Aging, National Institutes of Health, Bethesda, USA

76. Centre for Clinical Brain Sciences, University of Edinburgh, Edinburgh, United Kingdom
77. Primary Dementia Collaborative Research Centre - Assessment and Better Care, UNSW, Sydney, Australia
78. N.I. Vavilov Institute of General Genetics, Russian Academy of Sciences, Moscow, Russia
79. Division of Medical Genetics, Department of Biomedicine, University of Basel, Basel, Switzerland
80. Institute of Human Genetics, University of Bonn, Bonn, Germany
81. Institute of Neuroscience and Medicine (INM-1), Research Centre Jülich, Jülich, Germany
82. Department of Psychiatry and Psychotherapy, Charité Universitätsmedizin Berlin, CCM, Berlin, Germany
83. Clinical Research Branch, National Institute on Aging, Baltimore, USA
84. Medical and Molecular Genetics, Indiana University School of Medicine, Indianapolis, USA
85. University of Texas Health Science Center, San Antonio, USA
86. Biofunctional Imaging, Immunology Frontier Research Center, Osaka University, Osaka, Japan
87. Division of Genetics, Department of Medicine, Brigham and Women's Hospital, Boston, USA
88. Department of Psychiatry, University of Groningen, University Medical Center Groningen, Groningen, The Netherlands
89. Institute of Diagnostic Radiology and Neuroradiology, University Medicine Greifswald, Greifswald, Germany
90. Department of Psychology, VU University Amsterdam, Amsterdam, The Netherlands
91. Interfaculty Institute for Genetics and Functional Genomics, University Medicine Greifswald, Greifswald, Germany
92. Department of Psychiatry, Fujita Health University School of Medicine, Toyoake, Japan
93. Radiology, Mayo Clinic, Rochester, USA
94. FMRIB Centre, University of Oxford, United Kingdom
95. NICHD Brain and Tissue Bank for Developmental Disorders, University of Maryland Medical School, Baltimore, USA
96. University of Sussex, Brighton, United Kingdom
97. University College London, London, United Kingdom
98. University of Maryland, Catonsville, USA
99. Neuroscience Research Australia, Sydney, Australia
100. School of Medical Sciences, UNSW, Sydney, Australia
101. Columbia University Medical Center, New York, USA
102. Lymphocyte Cell Biology Unit, Laboratory of Immunology, National Institute on Aging, National Institutes of Health, Baltimore, USA
103. Centre for Advanced Imaging, University of Queensland, Brisbane, Australia
104. Ludwig-Maximilians-Universität, Munich, Germany
105. Department of Statistics & WMG, University of Warwick, Coventry, United Kingdom
106. Department of Genomics, Life & Brain Center, University of Bonn, Germany
107. Department of Psychiatry, Osaka University Graduate School of Medicine, Osaka, Japan
108. Institute of Psychiatry, King's College London, London, United Kingdom
109. Department of Neurology, University of Calgary, Calgary, Canada
110. Department of Clinical Neuroscience, University of Calgary, Calgary, Canada
111. Psychiatry and Human Behavior, University of California, Irvine, USA
112. Beth Israel Deaconess Medical Center, Boston, USA
113. Department of Neurology, Harvard Medical School, Boston, USA

114. Department of Psychiatry and Psychotherapy, University Heidelberg, Heidelberg, Germany
115. Department of Neuropathology, MRC Sudden Death Brain Bank Project, University of Edinburgh, Edinburgh, United Kingdom
116. Laboratory of Neuro Imaging, Institute for Neuroimaging and Informatics, Keck School of Medicine of the University of Southern California, Los Angeles, USA
117. Brain Resource Center, Johns Hopkins University, Baltimore, USA
118. Georgia State University, Atlanta, USA
119. The Scripps Research Institute, Jupiter, USA
120. Leiden University Medical Center, Leiden, The Netherlands
121. Neuroimaging Centre, University of Groningen, University Medical Center Groningen, Groningen, The Netherlands
122. Department of Psychiatry, Carver College of Medicine, University of Iowa, Iowa City, USA
123. Department of Neurobiology, Care Sciences and Society, Karolinska Institutet, Stockholm, Sweden
124. Research Resources Branch, National Institute on Aging, National Institutes of Health, Bethesda, USA
125. Faculty of Life Sciences, University of Manchester, Manchester, United Kingdom
126. Center for Integrative and Translational Genomics, University of Tennessee Health Science Center, Memphis, USA
127. Department of Anatomy and Neurobiology, University of Tennessee Health Science Center, Memphis, USA
128. Cognitive Genetics and Therapy Group, School of Psychology & Discipline of Biochemistry, National University of Ireland Galway, Galway, Ireland
129. Department of Clinical Genetics, Maastricht University Medical Center, Maastricht, The Netherlands
130. Department of Psychology, Center for Brain Science, Harvard University, Cambridge, USA
131. Karakter Child and Adolescent Psychiatry, Radboud university medical center, Nijmegen, The Netherlands
132. The Mind Research Network & LBERI, Albuquerque, USA
133. Department of ECE, University of New Mexico, Albuquerque, USA
134. Center for Translational Imaging and Personalized Medicine, University of California, San Diego, USA
135. Departments of Neurosciences, Radiology, Psychiatry, and Cognitive Science, University of California, San Diego, USA
136. Avera Institute for Human Genetics, Sioux Falls, USA
137. Neurology Division, Beaumont Hospital, Dublin, Ireland
138. Department of Neurology, Hopital Erasme, Universite Libre de Bruxelles, Brussels, Belgium
139. Department of Medical Genetics, Oslo University Hospital, Oslo, Norway
140. Janssen Research & Development, Johnson & Johnson, New Jersey, USA
141. Department of Psychiatry, University of Iowa, Iowa City, USA
142. Munich Cluster for Systems Neurology (SyNergy), Munich, Germany
143. University of Liverpool, Institute of Translational Medicine, Liverpool, United Kingdom
144. Institute of Clinical Chemistry and Laboratory Medicine, University Medicine Greifswald, Greifswald, Germany
145. Center for Imaging of Neurodegenerative Disease, San Francisco VA Medical Center, University of California, San Francisco, USA
146. Department of Child Psychiatry, Erasmus University Medical Centre, Rotterdam, The Netherlands
147. Department of Radiology, Erasmus University Medical Centre, Rotterdam, The Netherlands

148. Department of Clinical Neuroscience, Psychiatry Section, Karolinska Institutet, Stockholm, Sweden
149. Clinical Neuroimaging Laboratory, College of Medicine, Nursing and Health Sciences, National University of Ireland Galway, Galway, Ireland
150. Department of Psychiatry and Psychotherapy, HELIOS Hospital Stralsund, Germany
151. Molecular Research Center for Children's Mental Development, United Graduate School of Child Development, Osaka University, Osaka, Japan
152. Medical University of Lodz, Lodz, Poland
153. Department of Psychiatry, University of Oxford, Oxford, United Kingdom
154. King's College London, London, United Kingdom
155. Section of Gerontology and Geriatrics, Department of Medicine, University of Perugia, Perugia, Italy
156. Rotman Research Institute, University of Toronto, Toronto, Canada
157. Departments of Psychology and Psychiatry, University of Toronto, Canada
158. Departments of Physiology and Nutritional Sciences, University of Toronto, Canada
159. Neuropsychiatric Institute, Prince of Wales Hospital, Sydney, Australia
160. Department of Neuroimaging, Institute of Psychiatry, King's College London, London, United Kingdom
161. Biomedical Research Centre for Mental Health, King's College London, London, United Kingdom
162. Biomedical Research Unit for Dementia, King's College London, London, United Kingdom
163. Institute of Clinical Medicine, Neurology, University of Eastern Finland, Kuopio, Finland
164. Neurocentre Neurology, Kuopio University Hospital, Finland
165. Departments of Psychiatry, Neurology, Neuroscience and the Institute of Genetic Medicine, Johns Hopkins University School of Medicine, Baltimore, USA
166. Department of Epidemiology, Erasmus University Medical Centre, Rotterdam, The Netherlands
167. Laboratory of Epidemiology and Population Sciences, Intramural Research Program, National Institute on Aging, Bethesda, USA
168. Department of Neurology, Clinical Division of Neurogeriatrics, Medical University Graz, Graz, Austria
169. INSERM U897, University of Bordeaux, France
170. Department of Neurology, Boston University School of Medicine, Boston, USA
171. Framingham Heart Study, Framingham, USA
172. Department of Neurology, School of Medicine, University of Pittsburgh, Pittsburgh, USA
173. Department of Psychiatry, School of Medicine, University of Pittsburgh, Pittsburgh, USA
174. Department of Psychology, School of Medicine, University of Pittsburgh, Pittsburgh, USA
175. General Internal Medicine, Johns Hopkins School of Medicine, Baltimore, USA
176. Department of Radiology, Massachusetts General Hospital, Harvard Medical School, Boston, MA, USA.
177. Department of Neurology University of Washington, Seattle, USA
178. Institute of Molecular Biology and Biochemistry, Medical University Graz, Austria
179. Department of Neurology, Johns Hopkins University School of Medicine, Baltimore, USA
180. Department of Biostatistics, Boston University School of Public Health, Boston, USA
181. UMR5296 CNRS, CEA and University of Bordeaux, Bordeaux, France
182. Cardiovascular Health Research Unit, Department of Medicine, University of Washington, Seattle, USA
183. Icelandic Heart Association, Kopavogur, University of Iceland, Faculty of Medicine, Reykjavik, Iceland
